# Strong Acid-Mediated Proton Transfer via Water Tunneling Fosters Hydrogen Evolution Reaction on MoS_2_ Derivatives under Alkaline Conditions

**DOI:** 10.1021/acscatal.5c02610

**Published:** 2025-07-18

**Authors:** Matteo Pugliesi, Giulia Alice Volpato, Ida Ritacco, Giulia Tuci, Mattia Cattelan, Andrea Rossin, Yuefeng Liu, Lucia Caporaso, Matteo Farnesi Camellone, Giuseppe Santoriello, Elena Colusso, Stefano Agnoli, Giuliano Giambastiani

**Affiliations:** † Institute of Chemistry of OrganoMetallic Compounds, ICCOM-CNR and INSTM unit, Via Madonna del Piano 10, Sesto Fiorentino, Florence 50019, Italy; ‡ Department of Chemical Science, University of Padua and INSTM unit, Via Marzolo 1, Padova 35131, Italy; § Department of Chemistry and Biology “A. Zambelli″, University of Salerno, Via Giovanni Paolo II, Fisciano, Salerno 84084, Italy; ∥ Dalian National Laboratory for Clean Energy (DNL), Dalian Institute of Chemical Physics, Chinese Academy of Sciences 457 Zhongshan Road, Dalian 116023, China; ⊥ CNR-IOM Democritos and International School for Advanced Studies (SISSA), Via Bonomea 265, Trieste 34136, Italy; # Department of Industrial Engineering, University of Padua and INSTM unit, Via Marzolo 9, Padova 35131, Italy; ∇ Department of Chemistry “U. Schiff” (DICUS), University of Florence and INSTM unit, Via della Lastruccia 3-13, Sesto Fiorentino, Florence 50019, Italy

**Keywords:** chalcogenide surface engineering, hydrogen evolution reaction, water tunneling, computational models, acid−base water electrolytes

## Abstract

The surface functionalization of chemically exfoliated MoS_2_ (*CE*-MoS_2_) nanoflakes with Brønsted-acid end-capped aryl fragments adds an additional level of complexity to the comprehension of the correlation between the electron-donating strength of covalently grafted organic groups (Hammett parameter) and the HER performance of these hybrids. MoS_2_ nanoflakes decorated with aryl-sulfonic acids promote proton transfer via tunneling of H-species, where weaker benzoic acid groups fail. Thus, surface-engineered *CE*-MoS_2_ bearing sulfonic-acid end-capped dangling arms acts as an electrocatalyst that boosts HER kinetics even under an alkaline environment, where water dissociation represents the bottleneck of the process. Density functional theory (DFT) calculations have been used to corroborate experimental evidence and speculate on the role of acidic moieties with respect to water molecule *tunneling* and dissociation at the surface of the functionalized chalcogenide. The study represents a significant advance in the development of pH-insensitive electrocatalysts for HER.

## Graphical abstract

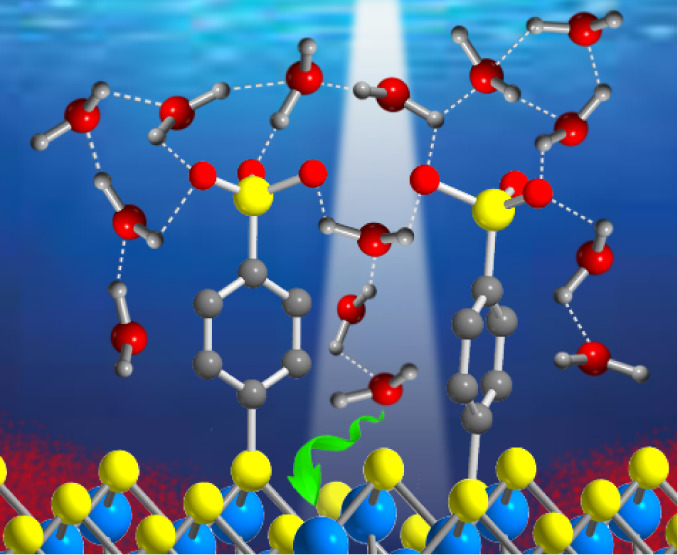

## Introduction

Hydrogen is globally recognized as one of the key vectors in charge of driving world energy transition.^1–3^ Nowadays, water electrolysis carried out using renewable energy sources is considered one of the most sustainable ways to produce hydrogen,^4,5^ although its widespread implementation is still limited by the lack of efficient and noncritical metal-based electrocatalysts working in a wide range of pH values. As far as the large-scale hydrogen production in industrial plants is concerned, the alkaline environment offers important technical and economic benefits with respect to acidic media; it allows the use of earth-abundant and inexpensive transition metal-based catalysts,^6^ avoids the use of expensive proton exchange membranes, and mitigates the slow electron-transfer kinetics of the oxygen evolution reaction (OER) at the anode of electrolyzers.^7,8^ In contrast with these positive features, high pH values slow down the kinetics of the hydrogen evolution reaction (HER)^9^ and the conversion efficiency of *state-of-the-art* electrocatalysts can be reduced by orders of magnitude moving from acidic to alkaline environment.^10^ Therefore, the development of highly efficient HER electrocatalysts capable of working under alkaline media is a challenging issue for chemists and chemical engineers. Yan and coworkers recently proposed the rational engineering of a Pt^δ−^ nanoparticle-based catalyst on oxygen-vacancy-enriched MgO nanosheets.^11^ Accordingly, MgO promoted H_2_O dissociation while H_3_O^+^ species accumulated electrostatically around the negatively charged Pt^δ−^ particles, hence mimicking the generation of an acidic microenvironment around the catalyst’s active sites even when the process was operated under alkaline conditions.

Developing low-cost HER catalysts for alkaline media also crosses another urgent issue of the catalysis and material science community: the removal of critical raw elements from catalyst compositions. In this regard, earth-abundant transition metal dichalcogenides (TMDs)—MoS_2_ being one of the most studied—have emerged as a promising class of cheap and effective HER electrocatalysts.^12,13^ In spite of a number of seminal contributions appeared in the literature to date, the HER performance of plain MoS_2_ electrocatalysts appears unsatisfactory, especially for processes operated under neutral and/or alkaline environments, where HER kinetics are largely limited by the slow water adsorption and dissociation process.^9,13^ A rational engineering of the MoS_2_ interface^14–17^ (including the use of a series of metal oxides/hydroxides acting as water dissociation promoters) has improved the efficiency of these hybrids in alkaline electrolytes by reducing the energy barriers associated with the initial water dissociation step and the subsequent H_2_ generation.^9,18,19^

Herein, we offer an original approach to boost alkaline HER kinetics with MoS_2_ nanoflakes through their chemical functionalization^20,21^ with Brønsted acid-capped aryl fragments [*i.e.*, aromatic carboxylic (Ar–COOH) and sulfonic (Ar–SO_3_H) acids]. The introduction of acidic dangling groups at the surface of MoS_2_ improves the material’s hydrophilicity and creates a surface microenvironment that boosts water tunneling and its activation/dissociation at the electrocatalyst surface, thereby equally increasing HER kinetics in both acidic and alkaline media.

## Materials and Methods

### General Considerations

All manipulations were carried out using standard Schlenk-type techniques under a nitrogen atmosphere. Chemically exfoliated (lithium intercalated) molybdenum disulfide (*CE*-MoS_2_; LiMoS_2_) and all other chemicals were provided by Merck and used as received without any further purification. Samples sonication was accomplished with an Elma S15 Elmasonic sonicator bath (37 kHz). To get a homogeneous water dispersion of the commercially provided *CE*-MoS_2_, a suspension of 10 mg of solid flakes was sonicated in about 45 mL of degassed Milli-Q water for 20 min at room temperature.

### Surface Functionalization of *CE*-MoS_2_ via Aryldiazonium Salt Chemistry

In a typical procedure, *CE*-MoS_2_ (40 mg) was suspended in 180 mL of Milli-Q water using a two-neck 250 mL flask and sonicated for 20 min at room temperature. Afterward, 0.25 mmol of the selected aniline (**1–2**), sodium nitrite (NaNO_2_, 0.3 mmol), and acetic acid (CH_3_COOH, 0.3 mmol) were added in sequence, and the suspension was additionally sonicated for 10 min at room temperature. The mixture was then heated at 60 °C for 14 h under vigorous magnetic stirring. After the mixture was cooled down to room temperature, the solid was almost entirely recovered by centrifugation before undergoing four successive sonication/washing cycles (2 × C_2_H_5_OH, ethanol and 2 × C_2_H_5_CO_2_CH_3_, ethyl acetate). The as-obtained solid materials (**3–4**) were recovered almost quantitatively before being dried at 50 °C under vacuum to constant weight and stored in air at room temperature, where they were stable without alterations for months. For each functionalized sample (**3–4**), a *blank sample* was prepared under identical reaction and workup conditions except for the use of the sodium nitrite. Each *blank* material recovered from the process workup resulted in an analytically pure MoS_2_ sample with no traces of contaminants, and it was processed accordingly. Blank samples were arbitrarily labeled as follows: ^b^ *CE*-MoS_2_.

### Materials Characterization

*FT-IR spectroscopy* was performed on a PerkinElmer Spectrum BX FT-IR spectrophotometer with a resolution of 1 cm^–1^ in the 400–4000 cm^–1^ range. Samples for analysis were prepared by mixing functionalized MoS_2_ hybrids with spectroscopic-grade KBr. *Thermogravimetric analysis* (TGA) was carried out under a N_2_ atmosphere (100 mL/min) on an EXSTAR Thermo Gravimetric Analyzer (TG/DTA) Seiko 6200 in the 40–700 °C range at a heating rate of 5 °C/min. *Elemental analyses* (EA) were performed on a Thermo FlashEA 1112 Series CHNS analyzer, and the average wt % values were calculated over three independent runs. *Acid–base titration* measurements were accomplished following previously setup procedures.^22^ In brief, 5 mg of **3** or **4** were suspended in a standardized NaOH solution (7 mL) and sonicated for 20 min. After being stirred at room temperature for 48 h, the suspension was filtered and three aliquots of the obtained clear solution were titrated with a standardized HCl solution. *Atomic resolution HAADF-STEM* images: High-angle annular dark field scanning transmission electron microscopy (HAADF-STEM) was performed on a JEM ARM200F instrument with a probe corrector at 200 kV. The sample was ultrasonicated in an ethanol solution and deposited by evaporative drop-casting on a copper grid covered with a holey carbon membrane. *X-ray photoelectron spectroscopy* (XPS) spectra were acquired in an ultrahigh-vacuum (UHV) chamber with a working pressure below 5 × 10^–9^ mbar, using a nonmonochromatic Al Kα line (*hν* = 1486.7 eV) and pass energy of 50 and 20 eV for survey and high-resolution spectra, respectively. The deconvolution into single chemical-shift components was carried out using Voigt functions. *Scanning electron microscopy* (SEM) was performed with a field emission source equipped with a GEMINI column (Zeiss Supra VP35), with an acceleration voltage of 5 kV by using the InLens high-resolution detector. *Static contact angle measurements* with the sessile droplet method were carried out using an Ossila contact angle goniometer by dispensing a 5 μL droplet of Milli-Q water onto the surface. Measurements were taken at least at two points on each surface. Samples for the analysis were prepared by the deposition of functionalized MoS_2_ hybrids on silicon substrates.

### Electrochemical Measurements

Electrochemical measurements were performed in sealed cells (glass for acidic electrolyte, polypropylene for alkaline) under N_2_ flow on an Autolab PG-STAT 204 potentiostat/galvanostat in a 3 electrode configuration, using a graphite rod as the counter electrode and either a saturated calomel electrode (SCE, 0.241 V vs SHE) or a mercury/mercury oxide electrode (MOE, 0.101 V vs SHE) as the reference electrode (RE) for acidic and alkaline electrolytes, respectively. The working electrode (WE) consisted of a 3 mm diameter glassy carbon electrode (GCE) coated with a film of the desired catalyst prepared as follows: 3 mg of the material of choice were suspended in 1.5 mL of DMF and sonicated for 30 min, then 21 μL (30% w/w with respect to catalyst) of 5% Nafion suspension in alcohol were added, followed by a further 30 min of sonication to yield a homogeneous 2 mg/mL ink. Immediately prior to ink deposition, glassy carbon electrodes were polished using diamond paste (from Struers: 3, 1, and 0.25 μm), sonicated in sequence in Milli-Q water and ethanol, and finally dried under N_2_ flow. 4 μL of ink was then carefully drop-cast on the GCE surface and the electrode was left to dry overnight under a protective dome.

Before each measurement, the electrolyte was purged by N_2_ bubbling for 20 min and then the N_2_ flow was maintained over the electrolyte surface during the analyses, while 5 min of N_2_ bubbling and stirring were used between measurements to remove dissolved H_2_. All the potentials were converted to the reversible hydrogen electrode (RHE) with the following equation (eq 1)­

$$E^{RHE}=E\;vs\;RE+E^{RE\;vs\;SHE}+0.059\cdot pH$$ 1

where *E* ^RHE^ is the potential in the RHE scale, *E* vs RE is the potential experimentally measured with the reference electrode, and *E* ^RE vs SHE^ is 0.241 and 0.101 V for SCE and MOE, respectively, as measured with the master electrode method; pH = 0.96 was used for the 0.1 M sulfuric acid electrolyte as calculated considering the second acid dissociation constant (Ka_2_ = 1.2 × 10^–2^ and as confirmed by measurement with a pH meter; pH = 13 was used for the 0.1 M KOH electrolyte. All the potentials were corrected for the ohmic drop (*iR* drop) due to the electrolyte resistance (Rs) between WE and RE that was estimated for each WE with a positive feedback method. Accordingly, voltammograms were corrected using the following equation (eq 2):

2 $$E^{iRcorr}=E^{RHE}‐i\cdot Rs$$

where *i* is the measured current and Rs is the ohmic resistance due to the electrolytic solution. Rs was typically around 35–50 Ω in 0.1 M sulfuric acid (^b^ *CE*-MoS_2_ ≈ 40 Ω, ${{\mathrm{MoS}}_{2}}^{{\mathrm{SO}}_{3}H}$ ≈ 35 Ω, MoS_2_^COOH^ ≈ 50 Ω) and 60–70 Ω in 0.1 M KOH (^b^ *CE*-MoS_2_ ≈ 60 Ω, ${{\mathrm{MoS}}_{2}}^{{\mathrm{SO}}_{3}H}$ ≈ 70 Ω, MoS_2_^COOH^ ≈ 70 Ω). Each measurement was run in duplicate or triplicate with different freshly prepared WEs to check for reproducibility.

Electrodes for chronopotentiometry were prepared on precut 1 × 3 cm strips of Toray paper cleaned by ultrasonication in sequence in Milli-Q water, acetone, and isopropanol (10 min each) and dried in N_2_. After that, they were masked with a Teflon tape to expose a circular active area of 0.196 cm^2^ and the ink was then deposited with a loading of 0.2 mg/cm^2^, allowed to dry, and further treated overnight under vacuum. Chronopotentiometry was performed in sealed glass or polypropilene cells under continuous N_2_ flow, with SCE and MOE as reference electrodes for acidic and alkaline electrolytes, respectively, and a carbon rod as the counter electrode. Due to the small volume of the cell (50 mL), during the 24 h chronopotentiometry a refill of 10 mL of predegassed electrolyte was operated after 12–16 h and toward the end of the experiment.

### Computational Details

The DFT simulations of water adsorption and the processes involved in the hydrogen evolution reaction (HER) on stoichiometric and S-defective 1T-MoS_2_ monolayers, in the presence and absence of Brønsted-acid ligands, were performed using the Quantum Expresso code^23^ employing the Perdew–Burke–Ernzerhof (PBE) exchange-correlation functional based on the generalized gradient approximation (GGA)^24^ and ultrasoft pseudopotentials.^25^ The spin-polarized Kohn–Sham equations were solved in the planewave pseudopotential framework, with the wave function basis set and the Fourier representation of the charge density constrained by kinetic energy cutoffs of 50 and 500 Ry, respectively. The calculations were carried out using a stoichiometric 6 × 6 supercell of the 1T-MoS_2_ monolayer, with 36 and 72 atoms of molybdenum (Mo) and sulfur (S), respectively, for a total of 108 atoms (Figure S9a). In the 1T phase, each Mo atom is octahedrally coordinated to six neighboring S atoms. The stoichiometric surface of 1T-MoS_2_ was functionalized with benzoic acid (Ar–COOH) and benzenesulfonic acid (Ar–SO_3_H) in their deprotonated forms, hence simulating a basic environment. The surface size was chosen to respect the experimental ratio of ligands to sulfur atoms (L/S ≈ 1:8–9). Since a high ligand coverage would generate symmetrical situations, the functionalized models considered the presence of only two ligands to better reproduce the experimental conditions. We are aware that it represents a simplification of the materials obtained experimentally but from a computational viewpoint it is not possible to capture the complexity of the real system. The optimized structures of the Brønsted acids coordinated to the 1T-MoS_2_ surface, 1T-MoS_2_@2PhSO_3_^–^ (136 atoms) and 1T-MoS_2_@2PhCOO^–^ (134 atoms), are reported in Figure S9b,c. Functionalization induces structural distortion in the 1T-MoS_2_ lattice, leading to its rearrangement into the 1T^#^ phase. This phase represents a distorted 1T-MoS_2_ surface that adopts structural motifs characteristic of the 1T′ phase, particularly near the functionalization sites, in agreement with previous theoretical studies.^26–28^ In this article, we do not explicitly distinguish between the two phases but use the 1T polytype for both cases.

We also evaluated the thermodynamic and kinetic variations of the hydrogen evolution reaction (HER) in the presence of vacant sites by removing sulfur atoms from the external layer of 1T-MoS_2_-based catalysts (V_S_). For both 1T-MoS_2_ and the functionalized systems, we considered configurations with one vacant site (V_S_) and two vacant sites (2 V_S_). In the case of 2 V_S_, the sulfur vacancies were positioned both close to each other and far apart. When ligands were present, the positions of the two sulfur vacancies were chosen based on the positions of the pendant acid sites.

From our calculations, it emerged that the position of the second sulfur vacancy did not lead to any significant electronic and/or structural rearrangements able to influence the processes involved in the HER. Therefore, in this work, we reported only the results derived from models with a single sulfur vacancy, ${1\mathrm{T‐}MoS}_{2,1V_{S}}$ (A′), ${1\mathrm{T‐}MoS}_{2,1V_{S}}$ @2PhSO_3_^–^ (B′), and ${1\mathrm{T‐}MoS}_{2,1V_{S}}$ @2PhCOO^–^ (C′) (see Figure S9a′–c′).

A separation of 26 along the *z* direction among consecutive monolayers was entered to prevent mutual influence with the periodic images, and during the optimizations all atoms in the supercell were allowed to fully relax. The Brillouin zone integration was performed on the Γ point only, and a Gaussian smearing with a width of σ = 0.02 eV was used in the calculations. All over the study, the long-range nonlocal effects, such as van der Waals (vdW) interactions, were considered by including the dispersion correction through the zero-damping DFT-D3 method of Grimme.^29^

Water adsorption energy on the different catalysts was computed using the following formula: *E*_ads_ = $E_{\mathrm{SURF}/H_{2}O}$ – (*E*_SURF_ + $E_{H_{2}O}$ ), where $E_{\mathrm{SURF}/H_{2}O}$ is the energy of the total system in which H_2_O is adsorbed on the 1T-MoS_2_ surface in the presence and/or in the absence of ligands and V_S_, *E*_SURF_ is the energy of the different supports, and $E_{H_{2}O}$ is the energy of the water molecule in the gas phase, respectively. A negative *E*_ads_ indicates stable adsorption, whereas a positive value indicates unstable adsorption.

The climbing image nudged elastic band (CI-NEB) method^30–32^ was used to evaluate the minimum-energy reaction paths (MEPs) and transition states (TSs) for H_2_ evolution on the 1T-MoS_2_-based catalysts. The Volmer reactions for the three catalysts, *i.e*., ${1\mathrm{T‐}MoS}_{2,1V_{S}}$ , ${1\mathrm{T‐}MoS}_{2,1V_{S}}$ @2PhSO_3_^–^, and ${1\mathrm{T‐}MoS}_{2,1V_{S}}$ @2PhCOO^–^, are reported in Schemes S1–S3.

We employed the Born–Oppenheimer AIMD simulations with the CP2K software package^33^ to investigate the ligands and surface V_S_-induced effects on the wettability of the 1T-MoS_2_ catalyst. To create the interface between the supports and water, the space between the slabs was entirely filled with 528 H_2_O molecules, which, considering the size of the systems, allows for a better description of the solvent phase in reasonable calculation times. The canonical NVT ensemble was employed with a target temperature of 350 K, which seems to better simulate the structural properties of water at room temperature,^34–36^ using the “canonical sampling velocity rescaling” thermostat proposed by Bussi and coworkers.^37^ An integration time step of 0.5 fs was adopted, and the mass of the hydrogen atoms was replaced with deuterium to allow for a larger time step. The AIMD simulations were run for a production phase of 30 ps, a more than enough duration for AIMD of complex systems like ours, which allows for meaningful and satisfying statistics.^34,38–41^

## Results and Discussion

The chemically exfoliated molybdenum disulfide (*CE*-MoS_2_) has been functionalized with two model acid-capped *p*-aniline derivatives via classical aryl-diazonium salts chemistry (Scheme 1).^20,42^ Thereby, Brønsted-acidic aryl moieties featured by different acid strengths [*e.g*., benzoic acid (p*K*_a_ = 4.19)^43^ and benzenesulfonic acid (p*K*_a_ = −2.70)][Bibr ref44] were introduced independently as MoS_2_ dangling arms, and the as-prepared samples were scrutinized as HER electrocatalysts in acidic and alkaline electrolytes. Details of the synthetic protocols and the materials characterization are outlined in the Supporting Information. At odds with related *CE*-MoS_2_ functionalization protocols from the literature,^42^ MoS_2_^COOH^ (**3**) and ${{\mathrm{MoS}}_{2}}^{{\mathrm{SO}}_{3}H}$ (**4**) were synthesized from the lithium-intercalated MoS_2_ with *in situ* prepared aryl-diazonium intermediates obtained upon reacting anilines **1** or **2** with a slight excess of NaNO_2_/CH_3_COOH. This synthetic route simplifies the functionalization scheme and lends itself to run *blank trials* (in the absence of NaNO_2_ as a promoter) to rule out the presence of unreacted or simply physisorbed reagents at the surface of the 2D material.^20^

**Scheme 1:**
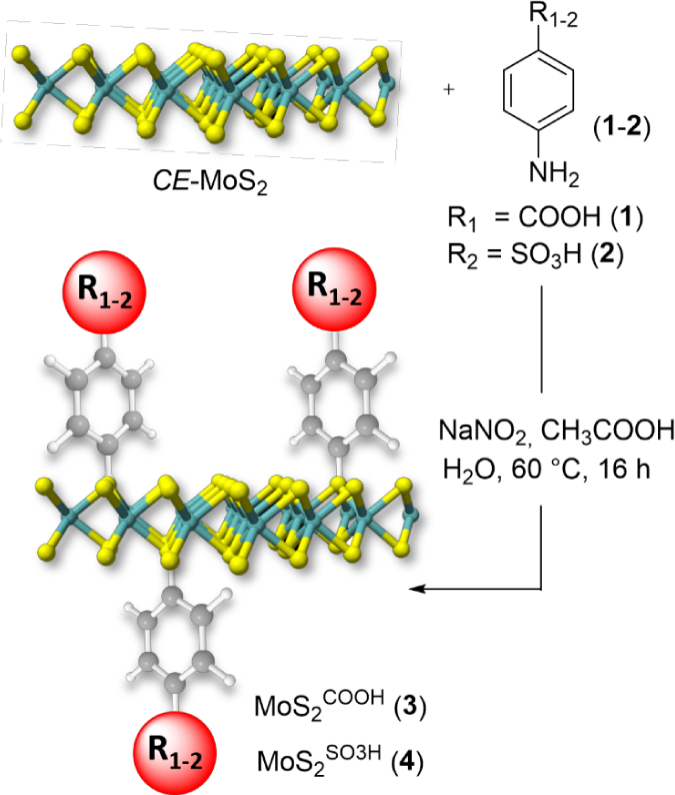
Exohedral Covalent Functionalization of *CE*-MoS_2_ via Aryl-Diazonium Salt Chemistry

Workup samples **3** and **4**, including their blank counterparts (^b^ *CE*-MoS_2_) and pristine *CE*-MoS_2_, underwent morphological and chemico-physical characterization. Selected characterization techniques were carried out on the most representative sample only [ ${{\mathrm{MoS}}_{2}}^{{\mathrm{SO}}_{3}H}$ (**4**)], according to its superior HER performance (*vide infra*). Evidence of the successful functionalization process is provided first by infrared (IR) spectroscopy (Figure S1). Distinctive stretching modes of carboxylic groups (ν = 1637 cm^–1^, C=O)^45^ and sulfonic moieties (ν = 1037 cm^–1^, S=O symmetric stretching),^46^ in **3** and **4**, respectively (not detectable in IR spectra of *blank* trials), validate the functionalization scheme and exclude—within the limits of IR sensitivity—any adventitious (not covalently bound) contamination of reagents. High-resolution X-ray photoelectron spectroscopy (XPS) of **3** and **4** at their C *1s* core photoemission level unveils the presence of H*C*sp^2^ moieties of the aryl rings (BE = 284.2 ± 0.2 eV),^21^ along with three distinctive components at 286.2, 288.5 ^47^ (Figure 1A) and 286.4 eV^48^ (Figure 1B), ascribed to *C*–S, *C*OOH and *C*–S*+C*_ipso_–SO_3_H, respectively. An adventitious C component at 284.6 eV also visible in the C *1s* spectrum of commercial *CE*-MoS_2_ (Figure S2A) indicates carbon contamination in the latter. Noteworthy, peak areas attributed to *C*–S*+C*OOH and *C*–S*+C*_ipso_–SO_3_H were comparable in accordance with the similar functionalization loading measured on **3** and **4** (see Table 1) and their ratios with respect to their corresponding H*C*sp^3^ components are perfectly in line with the expected functional groups (Scheme 1). The high-resolution photoemission spectrum at the S 2*p* core of **4** (Figure 1C) presents two typical components at 161.4 and 162.9 eV along with a shoulder at higher binding energies (168.1 eV; −SO_3_H group)^49^ due to sulfonic groups. Interestingly, both **3** and **4** exhibit a pronounced S 2*p* component at 162.9 eV with respect to pristine *CE*-MoS_2_ attributed to carboxylic and sulfonic aryl moieties chemically grafted to the chalcogenide. Covalent grafting via aryl diazonium salts occurs on the conductive and metastable 1T phase, which is stabilized, thus preventing the spontaneous phase evolution to the semiconductive 2H polymorph.^20,42,50^ The chemical grafting of aryl species bearing electron-withdrawing groups (EWG) causes a shift to higher BEs of the 1T S 2*p* component that overlaps with the 2H phase signal (Figure 1C and S2E).^50^ Similarly, Mo 3*d* photoemission spectra of **3** and **4** highlight a positive shift of the functionalized 1T phase compared to that of pristine *CE*-MoS_2_ (Figure S2C vs Figure S2D,F). Noteworthy, the Mo 3*d* high BE component accounts for about 15 at %, which aligns well with the content of −SO_3_H groups as measured from the S 2*p* spectrum of **4**. As expected, all MoS_2_ samples in this series exhibit an average S/Mo ratio of ≈1.9, which is consistent with the presence of sulfur vacancies. The latter likely originate from the harsh chemical exfoliation process of the bulk chalcogenide^51,52^ and are essential for activating water molecules in the HER under alkaline electrolytes (*vide infra*).

**Table 1:** Functional Group Loading as Estimated by Thermogravimetric Analysis (TGA), Elemental Analyses (EA) and Acid–Base Titration

	TGA^[^ ^a^ ^]^ (mmol/g)	EA^[^ ^b^ ^]^ (mmol/g)	acid–base titration^[^ ^c^ ^]^ (mmol/g)
MoS_2_^COOH^ (**3**)	0.76	0.98	0.83
${{\mathrm{MoS}}_{2}}^{{\mathrm{SO}}_{3}H}$ (**4**)	0.80	0.89	0.80

a Functional groups loading estimated from wt. loss % values reduced from weight loss of blank sample measured in the same temperature range.

b Values estimated from C wt % (Table S1) reduced by the contribution measured on blank counterparts. All values have been taken as the average result of three independent measures.

c Loading calculated as mean value of three independent runs.

**Figure 1:**
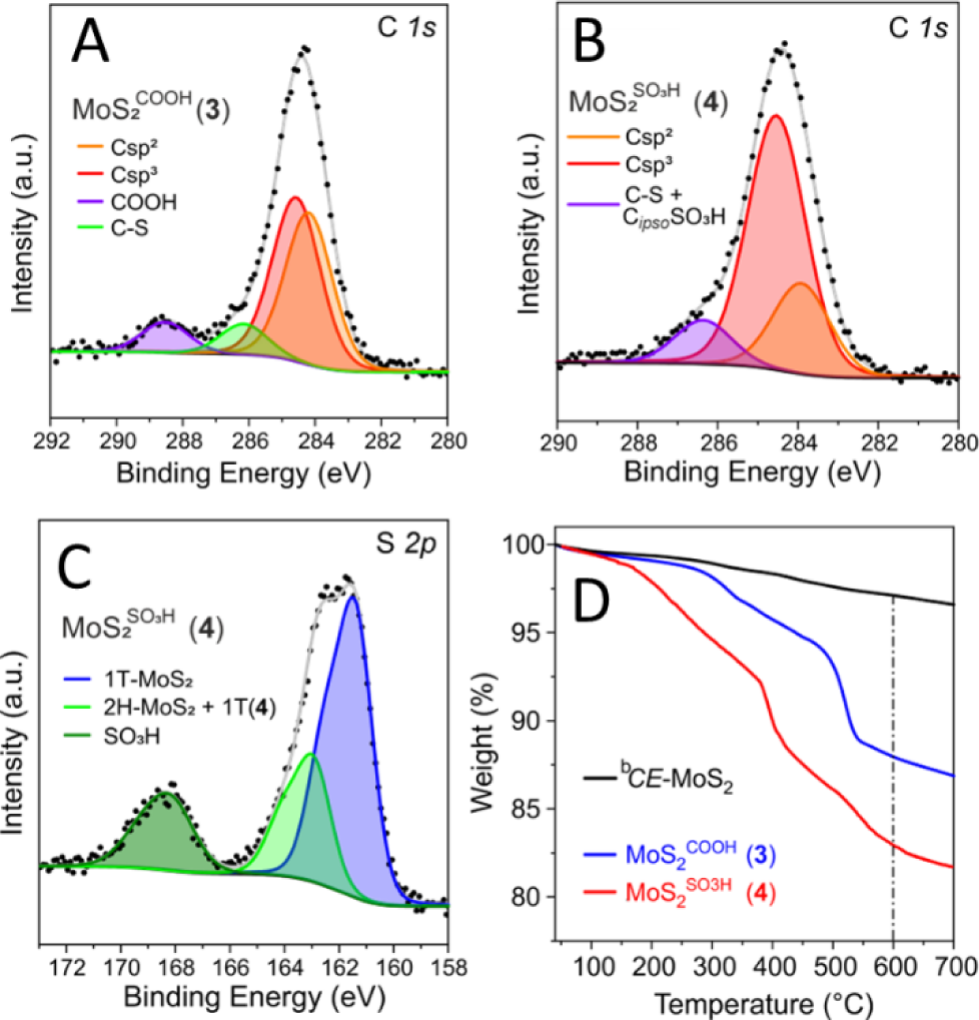
High resolution C *1s* core level region of **3** (A) and **4** (B). High resolution S *2p* core level region of **4** (C) and thermogravimetric profiles of **3** and **4** (D) along with that of pristine ^b^ *CE*-MoS_2_ (black curve): analysis conditions: 40–700 °C, 5 °C/min, N_2_ atmosphere (100 mL/min).

Scanning electron microscopy (SEM) of *CE*-MoS_2_ (Figure S3A,B) and **4** (Figure S3C,D) show similar micrometric lamellar structures featured by sharp edges crossing at 120° angles, without revealing any significant size alteration of MoS_2_ nanoflakes in line with the mild and virtually noninvasive conditions of the adopted functionalization protocol. Further details of the material’s morphology and microstructure were obtained from high-angle annular dark field scanning transmission electron microscopy (HAADF-STEM, Figure S4). Consistent with the XPS results, HAADF-STEM analysis of ${{\mathrm{MoS}}_{2}}^{{\mathrm{SO}}_{3}H}$ (**4**) and its pristine counterpart (*CE*-MoS_2_) reveals the coexistence of metallic 1T and semiconducting 2H domains with the former being predominant. This comparative analysis provides additional evidence that the covalent functionalization of MoS_2_ inhibits the spontaneous transformation of the metallic 1T polymorph into a more thermodynamically stable 2H phase.

The thermogravimetric analysis (TGA) of **3** and **4** along with that of pristine ^b^ *CE*-MoS_2_ was used to get a semiquantitative estimation of the samples’ functionalization loading (Figure 1D). **3** and **4** exhibited more pronounced weight losses compared to the pristine chalcogenide within the 40–600 °C temperature range. Thermograms at higher temperatures (>600 °C) revealed largely overlapping decomposition profiles, indicating that the thermal decompositions of grafted moieties were already complete within the aforementioned temperature range. All weight losses were normalized by subtracting those measured on the plain ^b^ *CE*-MoS_2_ (within the same temperature range), resulting in calculated functionalization loadings of 0.76 and 0.80 mmol/g for **3** and **4**, respectively (Table 1). Elemental analysis (CHNS-EA, Table S1) was used to confirm the extent of functionalization on **3** and **4**, as well as to ensure that no detectable amounts of merely physisorbed reagents remained after the samples’ workup. This analysis also confirmed the absence of N-containing molecules (*i.e.*, unreacted anilines **1** and **2**) and/or traces of sodium nitrite. It showed an appreciable increase of the C content in **3** and **4** due to the aryl grafting. Additionally, a significant increase in S wt %, attributed to the presence of sulfonate groups, was clearly observed in **4**. Overall, the EA data align well with the TGA analyses (Table 1) within the arbitrarily defined temperature decomposition range. Lastly, acid–base titration (see the Experimental Section for details) was employed to measure chemically accessible acidic groups on derivatives **3** and **4**. Titration data further confirmed the functionalization degree of **3** and **4** as estimated from TGA and EA data (≈ 0.8 mmol/g, Table 1).

^b^ *CE*-MoS_2_, **3**, and **4** were then employed as HER electrocatalysts in acidic [0.1 M H_2_SO_4_ (pH = 0.96)] and alkaline [0.1 M KOH (pH = 13)] electrolytes. To this end, we used a standard three-electrode cell equipped with a graphite rod and a saturated calomel electrode (SCE) as the counter and the reference electrodes, respectively. The working electrode was prepared by evaporative casting of a DMF/Nafion suspension of a selected chalcogenide sample on a ⌀ 3 mm-sized glassy carbon disk electrode to get an estimated catalyst loading of ≈ 0.11 mg/cm^2^. Linear sweep voltammograms (LSV) were acquired at a scan rate of 5 mV/s, and Tafel plots were derived from LSV at 0.5 mV/s. Table 2 provides a summary of all the figures of merit for the activity recorded using both MoS_2_ derivatives **3** and **4**, along with their respective blank counterparts. The analysis of curves in Figure 2, along with data in Table 2, underscores the crucial role of the acidic strength of the functional dangling arms in **3** and **4** with respect to their HER performance in acidic or basic electrolytes. Despite the comparable functionalization loading of **3** and **4** (Table 1), derivative **3** exhibits significantly poorer HER performance compared to plain ^b^ *CE*-MoS_2_ irrespective of whether the reaction environment is acidic or alkaline (Table 2, entries 1 vs 2 and 4 vs 5).

**Figure 2:**
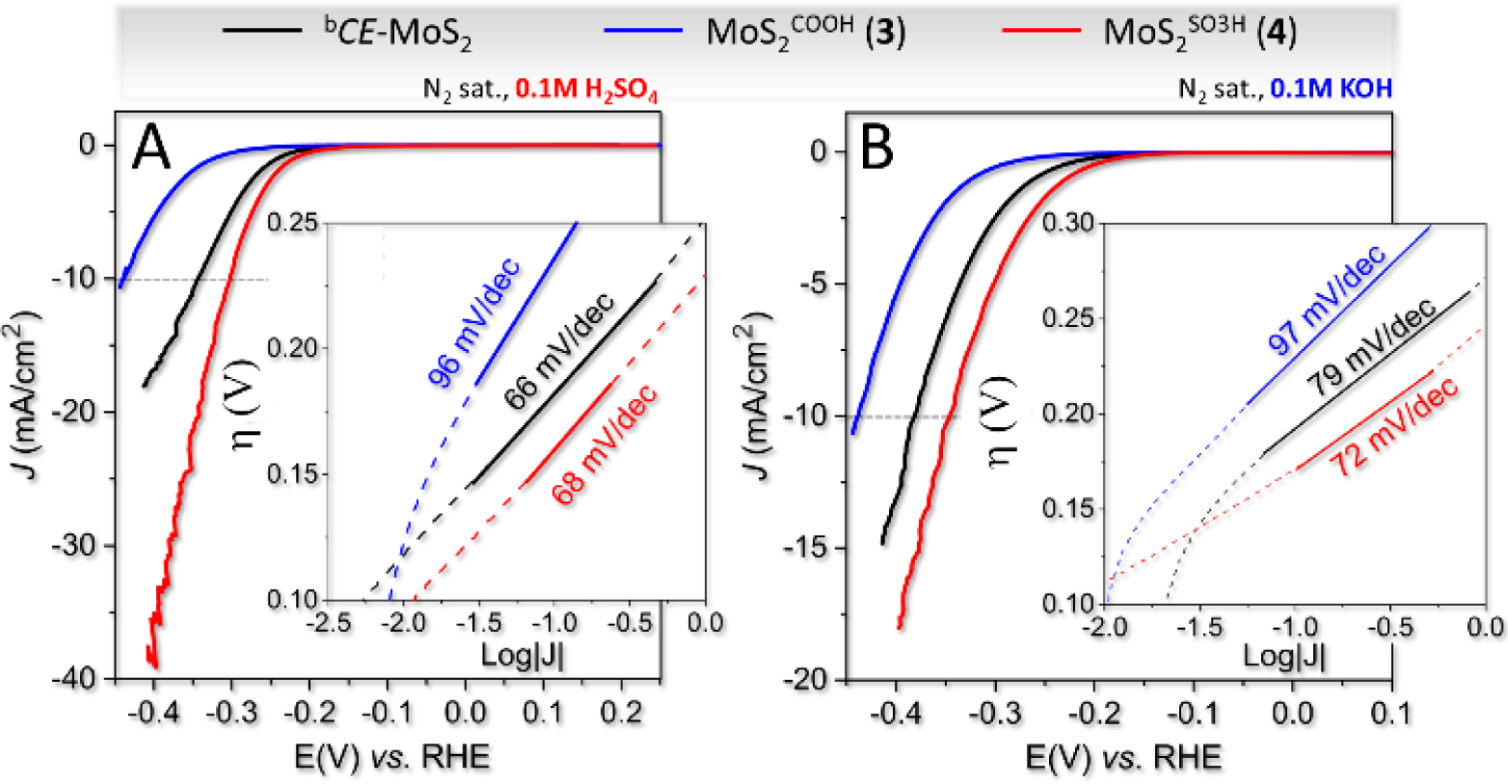
Electrocatalytic HER performance of different samples in acidic (A) and alkaline (B) electrolytes. *iR*-corrected LSVs were recorded in N_2_-saturated 0.1 M H_2_SO_4_ (A) and N_2_-saturated 0.1 M KOH (B) at a scan rate of 5 mV/s. The insets display Tafel analysis derived from *iR*-corrected LSVs obtained at a scan rate of 0.5 mV/s.

**Table 2:** Catalysts’ Performances in HER, Overpotential to Reach a Target Current Density Value, and Tafel Slope

			**η** (mV) @ ***X*** mA/cm^2^				
Entry	Catalyst	Electrolyte	***X*** = −0.1	***X*** = −2.0	***X*** = −5.0	***X*** = −10.0	Tafel slope(mV/dec)
1	^b^*CE*-MoS_2_	0.1 M H_2_SO_4_	172	265	301	343	66
2	MoS_2_^COOH^ (**3**)		236	387	454	-	96
3	${{\mathrm{MoS}}_{2}}^{{\mathrm{SO}}_{3}H}$ (**4**)		143	242	273	302	68
4	^b^*CE*-MoS_2_	0.1 M KOH	180	290	336	382	79
5	MoS_2_^COOH^ (**3**)		233	352	396	438	97
6	${{\mathrm{MoS}}_{2}}^{{\mathrm{SO}}_{3}H}$ (**4**)		155	256	301	346	72

Noteworthy, **4**, which contains the strongest sulfonic acid groups, exhibits a consistently positive overpotential shift (≈ 40 mV relative to ^b^ *CE*-MoS_2_ to achieve a current density of 10 mA/cm^2^) and irrespective of the pH of the electrochemical solution (Table 2, entries 1 vs 3 and 4 vs 6).

HER kinetics measured for both samples in acidic and basic environments follow similar, yet opposite trends. As shown in the Tafel curves of **3** (Figure 2), their slopes always account for worse kinetics compared to plain ^b^ *CE*-MoS_2_ (see also Table 2, entries 1 vs 2 and 4 vs 5).

On the other hand, Tafel slopes of **4** (Figure 2) demonstrate comparable or superior HER kinetics than those of ^b^ *CE*-MoS_2_, irrespective of the selected acid/base reaction medium (see Table 2, entries 1 vs 3 and 4 vs 6). This suggests an only moderate influence of pH on **4** HER performance and introduces a further level of complexity to the comprehension of the role of EWGs on the covalently grafted aryl moieties with respect to the HER performance of their MoS_2_ derivatives. Miller et al. first proposed a correlation between the electronic properties of covalently grafted aryl fragments and HER performance of their MoS_2_ hybrids.^50^ They showed that the overpotentials and Tafel slopes measured for their hybrid chalcogenides as HER systems operated in acidic electrolytes correlated with the Hammett parameter (surface dipole) of the chemically grafted aryl groups. According to this theory, compounds **3** and **4** should behave similarly given their similar functionalization loading and Hammett value of the organic moieties in a strong acidic environment (pH = 1; + 0.45 for **3** and +0.35 for **4**, respectively).^53^ However, this prediction clearly contrasts with the different aptitude of **3** and **4** to promote HER in strongly acidic (Table 2, entry 2 vs 3) and alkaline (pH = 13). The attempt to rationalize HER performance of **3** and **4** on the basis of materials’ surface dipoles failed. While strong sulfonic acid groups are deprotonated whatever the pH of the electrolyte and the material surface dipole does not change from low to high pH values (*i.e*., + 0.35), benzoic acid groups of **3** are protonated at pH ≈ 1 and quantitatively deprotonated (*i.e.*, carboxylate −COO^–^) at high pH values. Accordingly, their Hammett parameter varies from +0.34 at pH = 1 to 0.00 at pH = 13.^53^ In contrast, **3** does not improve its performance from strong acidic to strong alkaline conditions.^50^ This implies that the Hammett parameter cannot be invoked as the unique descriptor of HER with MoS_2_ hybrids. Evidence suggests that the greater the strength of dangling acidic groups [−SO_3_H (**4**) vs −COOH (**3**)], the better the HER performance of MoS_2_ derivatives, regardless of the acidic or basic nature of the electrolyte medium. This prompted us to claim strong surface acids as key promoters of HER kinetics under strong alkaline conditions. They were thought to facilitate the water dissociation step, traditionally conceived as the kinetic *bottleneck* for HER in high pH media. Strongly acidic groups impart superior wettability to the hydrophobic chalcogenide and create catalytic microenvironments where HER takes place conveniently, regardless of the electrolyte solution pH, while enabling rapid release of H_2_ from the sample surface.^9^ In acidic media, higher material wettability results in a greater H_3_O^+^ concentration gradient at the chalcogenide surface that facilitates the electrosorption of solvated protons (H*) through the Volmer–Heyrovsky steps (Figure S5A).^54,55^ Therefore, the presence of a strongly acidic reservoir covalently bonded to MoS_2_ accelerates this process. Conversely, under alkaline conditions, the initial and rate-limiting step for the hydrogen evolution reaction (HER) is water dissociation (Volmer–Heyrovsky steps, Figure S5B), which supplies H* to MoS_2_ and initiates the process; enhancing the water dissociation step thus significantly improves catalyst performance.^56^ Sulfone groups in **4** have the ability to establish strong hydrogen bonds with water molecules of the electrolyte solution. This facilitates what we defined as the water tunneling to the hydrophobic chalcogenide surface where water dissociation takes place, enabling proton transfer.^57,58^ As a result, the aryl-sulfone-mediated proton transfer promotes HER in alkaline environments, maintaining consistent kinetics and comparable (positive) overpotential shifts (vs ^b^ *CE*-MoS_2_) in both strongly basic and strongly acidic media. Accordingly, **4** stands out among the most effective MoS_2_-based hybrid materials for HER under acidic conditions (see Table S2).^50,59–63^ It also represents the first instance of a chemically functionalized chalcogenide serving as an HER electrocatalyst, exhibiting nearly pH-independent process kinetics. The variation in HER performance of **3** vs **4** is tentatively ascribed to differences in the acidic strength of acid groups on dangling aryl arms as well as their differing aptitude to form strong hydrogen bonds with the solvent, thereby enhancing material wettability and tunneling of H-species. As Figure S6 shows, contact angle measurements on pristine MoS_2_ and functionalized samples strongly support this assumption, clearly revealing the superior hydrophilic nature of the aryl-sulfone-decorated material **4** (θ = 23°) compared to the others (pristine MoS_2_: θ = 54° and **3**: θ = 88°).

Stability tests were finally conducted on the best-performing sample **4**, under both high and low pH conditions. As Figure S7 shows, the catalyst exhibited about a 5% and 10% reduction in the HER overpotential to achieve a current density of 10 mA/cm^2^, after being operated for 24 h in alkaline and acidic electrolytes, respectively. To further confirm the stability of the sample throughout the HER process, XPS analysis was repeated on the recovered material. Except for the C 1*s* and S 2*p* core regions, which were not reported due to evident Nafion interference, the Mo 3*d* peaks of the freshly prepared and used electrocatalyst exhibited nearly identical spectroscopic features, regardless of the electrolyte pH used (Figure S8).

The role of aryl-sulfonic groups in **4** as promoters for the tunneling and activation of water molecules in HER under alkaline conditions was ultimately elucidated through a comprehensive series of computational studies. These studies also served to give a rationale to the lower water affinity and worse electrochemical behavior of derivative **3** in HER. Density functional theory (DFT) modeling was performed on a stoichiometric (6 × 6) supercell of a 1T-MoS_2_ monolayer to which aryl-sulfonic or benzoic fragments were added in quantities matching the experimental findings (1 Ar^X^ group for every 8–9 S atoms). The systems were then simplified by reducing the number of dangling units to only two, thereby excluding fragments that generated symmetric scenarios. Accordingly, 1T-MoS_2_ (**A**), 1T-MoS_2_@2PhSO_3_^–^ (**B**), and 1T-MoS_2_@2PhCOO^–^ (**C**) were modeled along with their sulfur-defective (V_S_) counterparts [ ${1\mathrm{T‐}MoS}_{2,1V_{S}}$ (**A′**), ${1\mathrm{T‐}MoS}_{2,1V_{S}}$ @2PhSO_3_^–^ (**B′**), and ${1\mathrm{T‐}MoS}_{2,1V_{S}}$ @2PhCOO^–^ (**C′**)] (Figure S9). Defective structures were created by removing one sulfur atom (V_S_ = sulfur vacancy) from the outer material’s surface. The sulfur vacancy was rationally located near the aryl-grafted S-site because any other distal position far from the ligand would result in a catalytic behavior similar to **A′**. Defective sites, such as V_S_, are mandatory for the water activation/dissociation step to take place. Indeed, H_2_O adsorption at the surface of a stoichiometric monolayer [with (**B**, **C**) or without (**A**) surface-grafted aryl-arms] is thermodynamically unfavorable, hence repulsive (Figure S10). On the other hand, H_2_O adsorption is stabilized in all sulfur-defective models exhibiting coordinatively unsaturated Mo-sites (Figures S11). Notably, water adsorption energy (*E*_ads_) was further stabilized by ≈ 0.5/0.6 eV in **B′** and **C′** compared to the bare and defective **A′** monolayer (Figure 3 and Scheme S3). This extra stabilization is due to covalently bonded organic ligands, which induce structural distortions in the 1T-MoS_2_ lattice, leading to a 1T-to-1T^#^ phase transition.^26–28^ Consequently, the exposure of metal sites at the S-vacancy is enhanced, enabling Mo atoms to interact more easily with the oxygen of water molecules (Figure S12).

**Figure 3:**
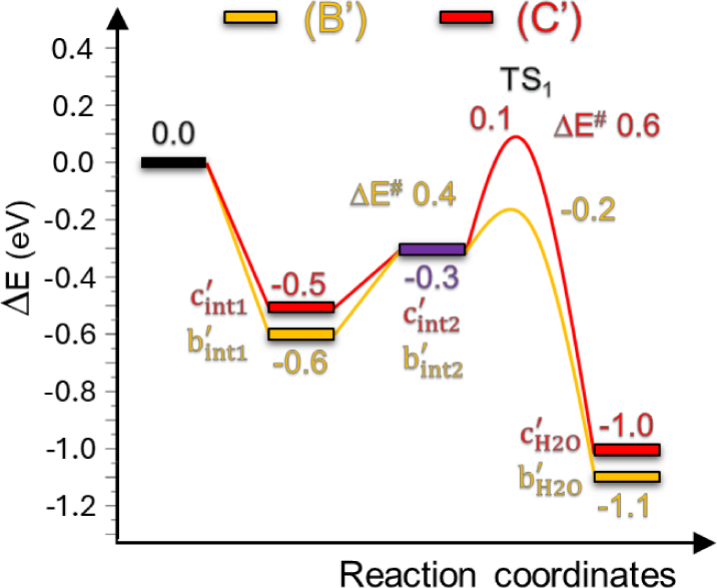
Reaction coordinates for the H_2_O tunneling at the Volmer step in alkaline environment [H_2_O adsorption at V_S_ **B′** ( ${1\mathrm{T‐}MoS}_{2,1V_{S}}$ @2PhSO_3_^–^, orange line) and **C′** ( ${1\mathrm{T‐}MoS}_{2,1V_{S}}$ @2PhCOO^–^, red line)] from climbing image nudged elastic band (CI-NEB) simulations.

The water dissociation, which is relevant in the Volmer step of HER in alkaline electrolytes (Figure S5), was then examined specifically for the three defective models: **A′**, **B**′, and **C′**. Reaction pathways and activation energies (TS_1_) for water adsorption on **B′** and **C′**, as determined through climbing image nudged elastic band (CI-NEB) simulations, are detailed in Figure 3 and Scheme S1. Regardless of the type of acid-capped aryl group, surface-grafted organic moieties promote water tunneling to V_S_, where its adsorption (and subsequent dissociation, as discussed below) occurs. Two H-bond-stabilized intermediates (b′_int1_/b′_int2_ and c′_int1_/c′_int2_) are formed from one water molecule and two neighboring aryl arms (Figures 3, S13 and Scheme S1). Notably, the optimized geometries for **B′** and **C′** exhibit the lowest energy structures when a meta-hydrogen (H_meta_) of an aryl fragment forms an agostic interaction with the coordinatively unsaturated Mo-site at the V_S_ (Figure S14).

The stability of the Mo–H_meta_ agostic interaction weakens as the Hammett parameter of the aryl fragments increases. This results in a lower energy barrier in **B′** related to the coordination exchange between the aryl-H_meta_ and the oxygen of an incoming water molecule at the Mo-site in V_S_ (Figure 3, ${b’}_{H_{2}O}$ and ${c’}_{H_{2}O}$ ). Consequently, the higher energy barrier (TS_1_) observed in **C′** explains the worse electrochemical performance of **3** vs **4**. This also offers a rationale for the differing water affinity and wettability observed between the two electrocatalysts (Figure S6). Once water adsorption occurs, the subsequent dissociation step follows a similar energetic pathway regardless of the defective model, including the unfunctionalized **A′** (Schemes S2 and S3). The data consistently underscore the importance of dangling aryl fragments and their associated surface dipoles in enabling water tunneling, which deeply impacts the HER kinetics of the electrocatalyst **4** in alkaline water electrolytes.

*Ab initio* molecular dynamics (AIMD) was carried out on all defective and gas-phase optimized configurations as a proof of concept. These simulations demonstrate the enhanced wettability of **B′**, which is linked to the hybrid’s intrinsic capability to swiftly facilitate the exchange between agostic aryl-H_meta_ and H_2_O coordination at the Mo-site in V_S_. On the other hand, **C′** exhibits a distinct hydrophobic nature, which aligns well with the experimental findings (Figure S14 and AIMD movies on Supporting Information).

In summary, chemically grafting strongly acidic end-capped aryl fragments onto *CE*-MoS_2_ stabilizes the electrochemically active 1T metallic phase and creates an optimal surface microenvironment for carrying out HER. These grafted moieties enhance HER kinetics by facilitating water tunneling to the chalcogenide surface, even under alkaline conditions, where water adsorption/dissociation typically represents the rate-limiting step.

## Supplementary Material







## Notes

Infrared spectra, high resolution C *1s*, S *2p* and Mo *3d* XPS spectra, elemental analyses, SEM micrograph, HAADF-STEM images, proposed reaction pathway for HER under acidic and alkaline conditions, contact angle measurements, durability tests, optimized structure of the stochiometric and S-defective catalysts, optimized geometries of the water molecule adsorption on the stochiometric and S-defective catalysts, reaction pathways of the water adsorption at the Volmer step in alkaline environment on the S-defective functionalized and undecorated catalysts (PDF)
(MP4) Ab initio molecular dynamics simulations of solvation effects on 1T-MoS2,1Vs@2PhCOO- (C’) catalyst
(MP4) Ab initio molecular dynamics simulations of solvation effects on 1T-MoS2,1Vs@2PhSO3- (B’) catalyst
