# Supplementary material for: Strong Acid-Mediated Proton Transfer via Water Tunneling Fosters Hydrogen Evolution Reaction on MoS2 Derivatives under Alkaline Conditions
Source: ACS Catal. 2025 Jul 18;15(15):13278–87. doi: 10.1021/acscatal.5c02610 (PMC12323386; doi:10.1021/acscatal.5c02610)
Supplement: Supplementary file 1 [file cs5c02610_si_001.pdf]

Strong Acid Mediated Proton Transfer via Water Tunneling Fosters Hydrogen Evolution Reaction on MoS_2 Derivatives Under Alkaline Conditions

Matteo Pugliesi,[§,‡] Giulia Volpato,[δ,‡] Ida Ritacco,[ω,‡] Giulia Tuci,[§,*] Mattia Cattelan,[δ,*] Andrea Rossin,[§] Yuefeng Liu,[φ] Lucia Caporaso,[ω,*] Matteo Farnesi Camellone,[π] Giuseppe Santoriello,[ω] Elena Colusso,[γ] Stefano Agnoli [δ,*] and Giuliano Giambastiani [ξ,§,*]

[§] Institute of Chemistry of OrganoMetallic Compounds, ICCOM-CNR and INSTM unit. Via Madonna del Piano 10 – 50019, Sesto Fiorentino, Florence, Italy. E-mail: giulia.tuci@iccom.cnr.it

[δ] Department of Chemical Science of the University of Padua and INSTM unit. Via Marzolo 1 - 35131 Padova, Italy. E-mail: mattia.cattelan@unipd.it, stefano.agnoli@unipd.it

[ω] Department of Chemistry and Biology "A. Zambelli", University of Salerno. Via Giovanni Paolo II, 84084 Fisciano (SA), Italy. E-mail: lcaporaso@unisa.it

[φ] Dalian National Laboratory for Clean Energy (DNL), Dalian Institute of Chemical Physics, Chinese Academy of Sciences. 457 Zhongshan Road, 116023 Dalian, China.

[π] CNR-IOM Democritos and International School for Advanced Studies (SISSA). Via Bonomea 265, Trieste, 34136, Italy.

[γ] Department of Industrial Engineering of the University of Padua and INSTM unit. Via Marzolo 9 – 35131 Padova, Italy.

[ξ] Department of Chemistry "U. Schiff" (DICUS), University of Florence and INSTM unit. Via della Lastruccia, 3-13, 50019, Sesto Fiorentino, Italy. E-mail: giuliano.giambastiani@unifi.it

[‡] These authors have equally contributed to the work

Contents:

Figure S1 Infrared spectra of $\mathbf{MoS_2^{COOH}}$ (**3**) and $\mathbf{MoS_2^{SO3H}}$ (**4**) ……………………….……. S3

Figure S2 High resolution C *1s*, S *2p* and Mo *3d* XPS spectra of *CE*-MoS_2, MoS_2^{COOH} (**3**) and MoS_2^{SO3H} (**4**)……………………...……………………………..……………………… S4

Table S1 CHNS Elemental analyses …………………………………………………...….. S5

Figure S3 SEM micrograph of *CE*-MoS_2 and MoS_2^{SO3H} …………………………………... S5

Figure S4 HAADF-STEM images of *CE*-MoS_2 and MoS_2^{SO3H} (**4**) ………………………... S6

Figure S5 Proposed reaction pathway for HER under acidic and alkaline conditions ……... S7

Figure S6 Contact angle measurements on *CE*-MoS_2, MoS_2^{COOH} (**3**) and MoS_2^{SO3H} (**4**) …… S7

Table S2 HER promoted by MoS_2 nanosheets functionalized with organic groups ………... S8

Figure S7 Durability tests for MoS_2^{SO3H} (**4**)…………………………………………….. S9

Figure S8 High resolution Mo *3d* XPS spectra of MoS_2^{SO3H} (**4**) recovered after HER electrocatalytic tests………………………………………………………………………… S10

Figure S9 Optimized structure of the stochiometric and S-defective catalysts …………… S11

Figure S10 Optimized geometries of the water molecule adsorption on the stochiometric catalysts ……………………………………………………………………………………… S11

Figure S11 Optimized geometries of the water molecule adsorption on the S-defective catalysts ……………………………………………………………………………………… S12

Figure S12 Top view of the optimized structure of the 1T-$MoS_{2,1Vs}$ catalyst……………… S12

Scheme S1 Reaction pathways of the water adsorption at the Volmer step in alkaline environment on the S-defective functionalized catalysts ………………………………… S13

Scheme S2 Reaction pathways of the water adsorption and dissociation at the Volmer step in alkaline environment on the S-defective functionalized catalysts……………………… S13

Scheme S3 Reaction pathway of the water adsorption and dissociation at the Volmer step in alkaline environment on the S-defective undecorated catalysts……………………….. S14

Figure S13 Optimized geometries of b'_{int1}/b'_{int2} and c'_{int1}/c'_{int2} intermediates ………………………………………………………………………………………… S16

Figure S14. Energy differences between the two possible geometries for 1T-$MoS_{2,1Vs}$@2$PhSO_3^-$ and 1T-$MoS_{2,1Vs}$@2$PhCOO^-$………………………………………… S17

References S17

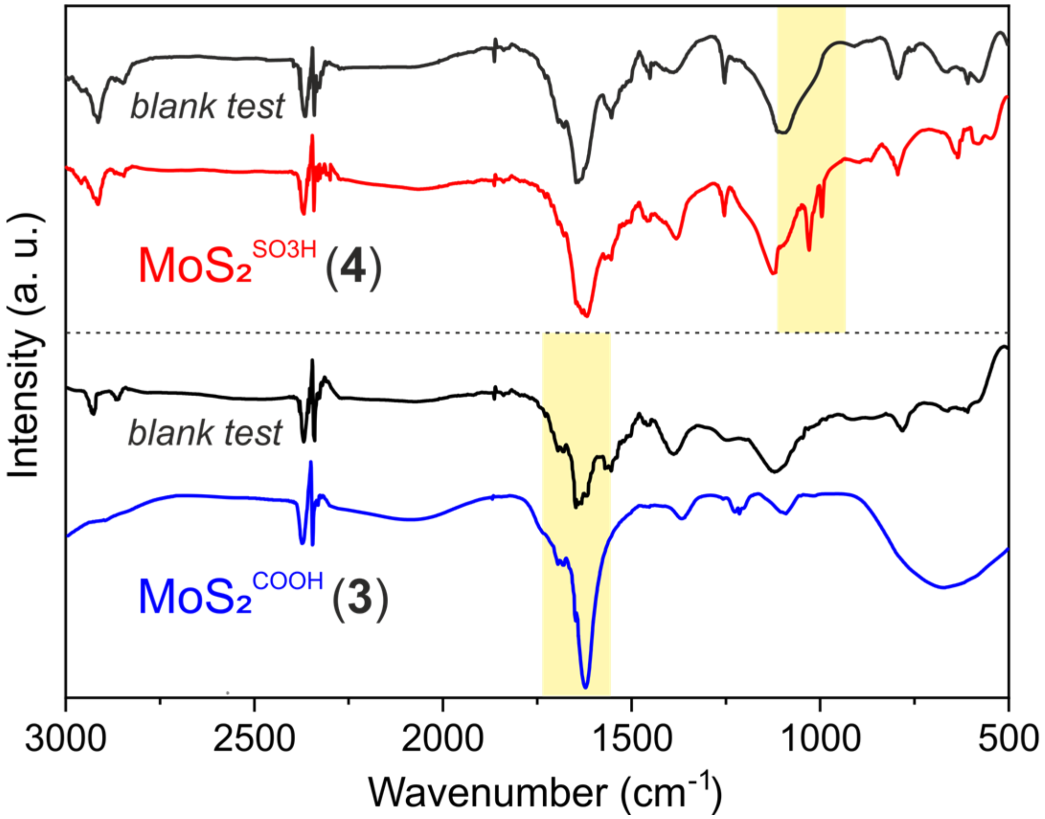

Figure S1. Infrared spectra of MoS_2^{COOH} (**3**) (bottom side) and MoS_2^{SO3H} (**4**) (upper side) at comparison with respective *blank* samples.

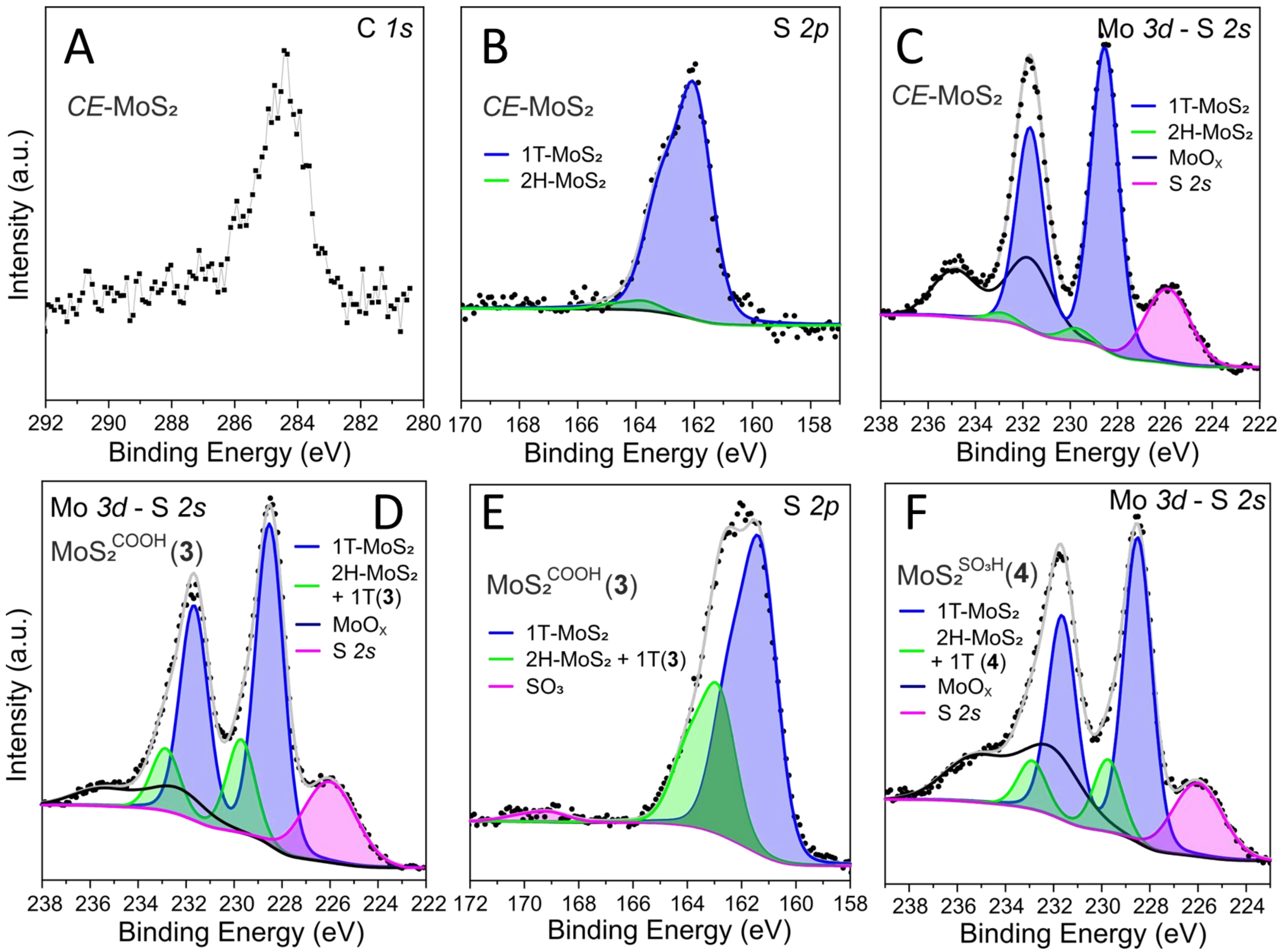

Figure S2. High resolution C 1*s*, S *2p* and Mo *3d* XPS lines of *CE*-MoS_2 (**A**-**B**-**C**), Mo *3d* and S *2p* XPS lines of MoS_2^{COOH} (**3**) (**D**-**E**) and Mo *3d* of MoS_2^{SO3H} (**4**) (**F**).

Table S1. CHNS Elemental analyses

	N wt.% [a]	C wt.% [a]	H wt.% [a]	S wt.% [a]
CE-MoS_2	-	1.63	0.76	22.06
MoS_2^{COOH} (**3**)	-	10.24	0.97	24.53
blank test	-	1.96	0.45	23.12
MoS_2^{SO3H} (**4**)	-	8.59	1.08	29.00
blank test	-	2.16	0.36	23.39

[a] average values over three independent measurements

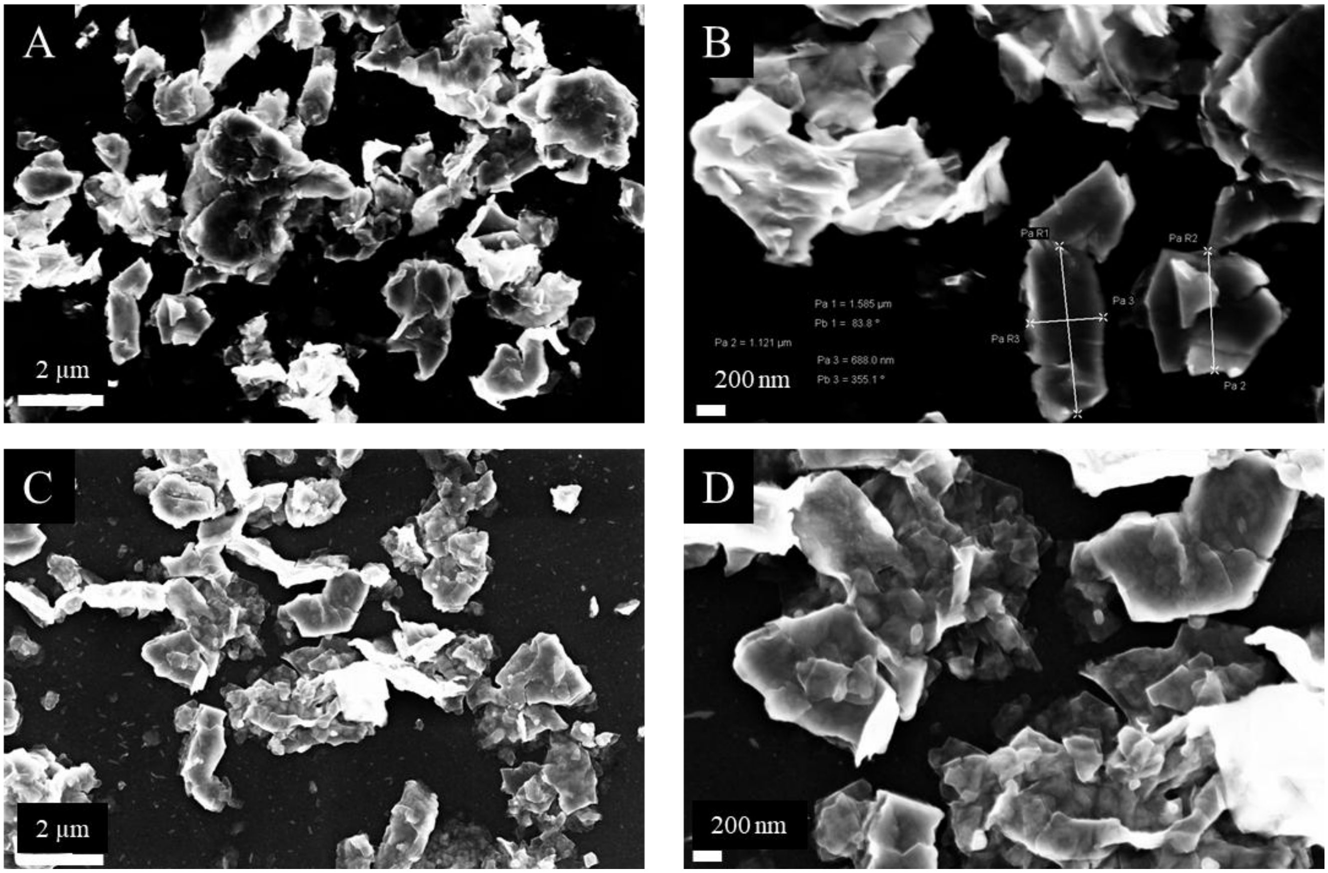

Figure S3. SEM micrographs of *CE*-MoS_2 (**A**-**B**) and MoS_2^{SO3H} (**4**) (**C**-**D**) at different magnifications.

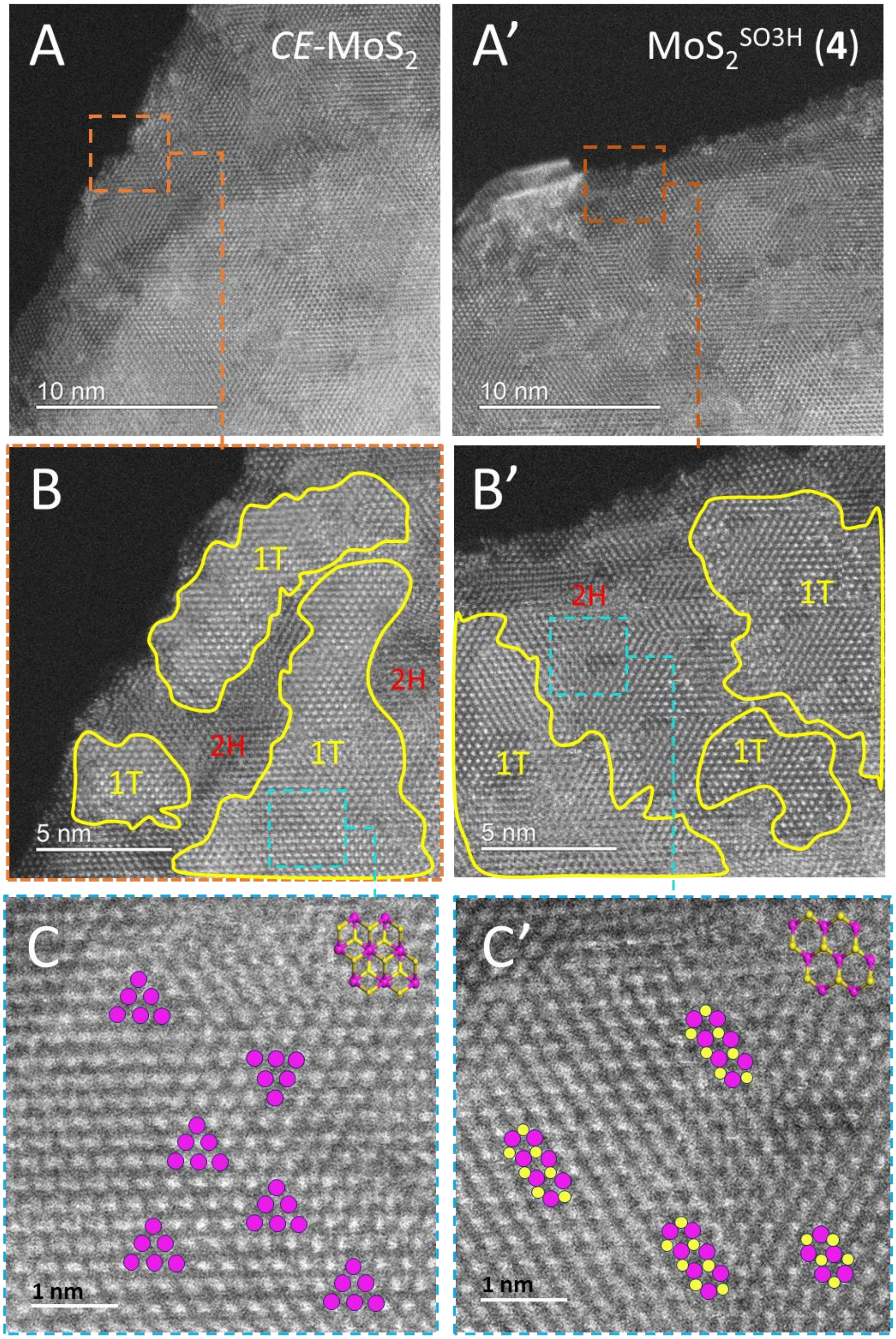

Figure S4. HAADF-STEM images of *CE*-MoS_2 (**A-C**) and MoS_2^{SO3H} (**4**) (**A'-C'**) at different magnifications. Figures S4B and S4B' highlight the 1T and 2H domains of *CE*-MoS_2 and MoS_2^{SO3H} (**4**), respectively. Magnifications in S4C and S4C' illustrate the , respectively. Atomic arrangement of 2H phase and 1T phase are conventionally labeled by colored dots (orange dots represent sulfur atoms while pink dots represent molybdenum atoms).

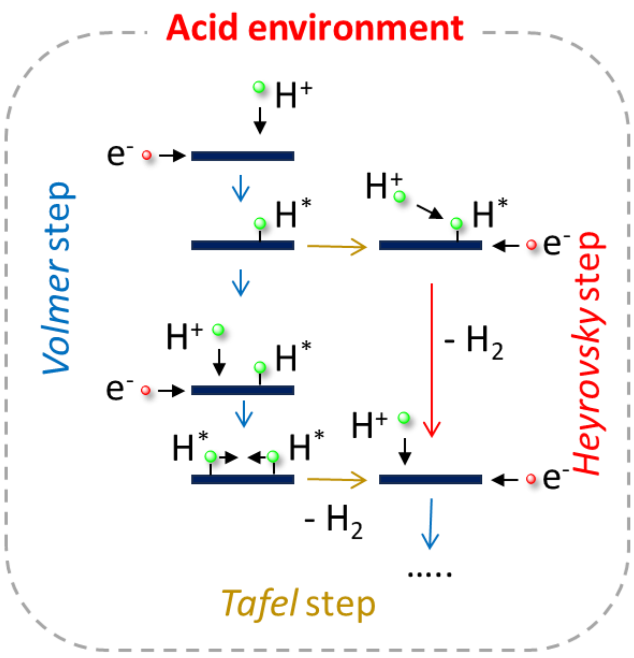

Figure S5. Proposed reaction pathway for the hydrogen evolution reaction (HER) under acidic (left-side hand) and alkaline (right-side hand) conditions.

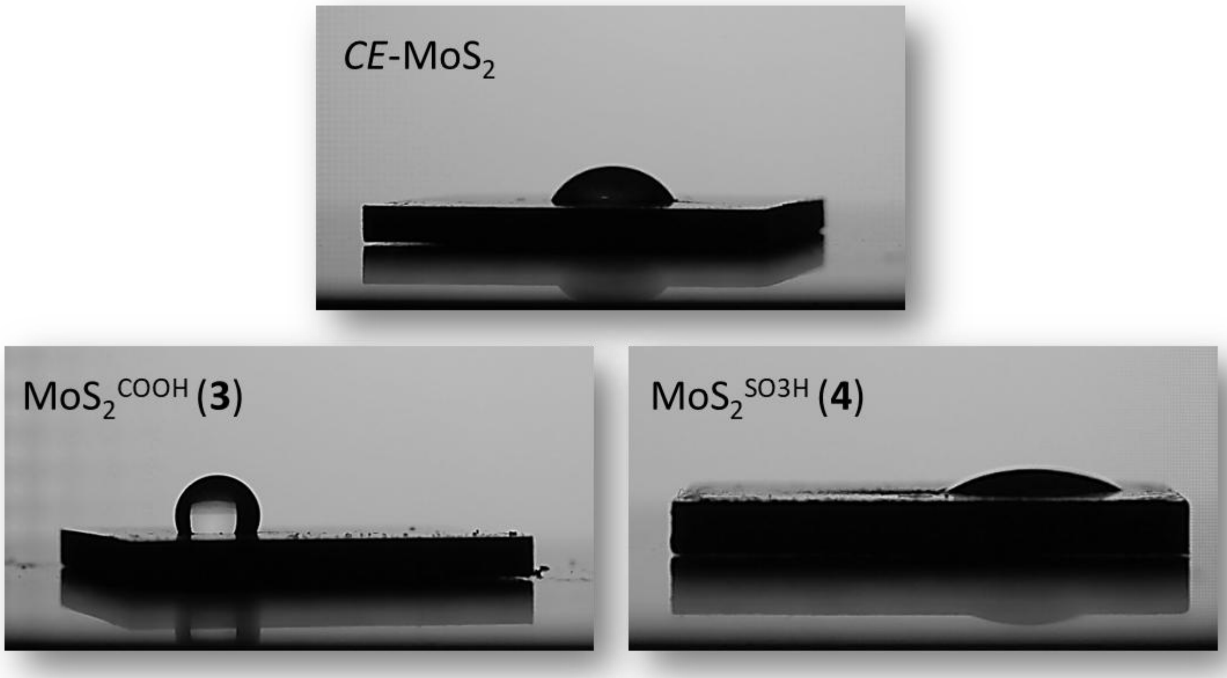

Figure S6. Images of contact angle of water on *CE*-MoS_2, MoS_2^{COOH} (**3**) and MoS_2^{SO3H} (**4**). The contact angles for the sessile droplet of water were: 54° (*CE*-MoS_2), 88° (MoS_2^{COOH}, **3**) and 23° (MoS_2^{SO3H}, **4**).

Table S2. HER promoted by MoS_2 nanosheets functionalized with discrete organic groups

Catalyst	Electrolyte	η (mV) @-10 mA/cm^2	Tafel slope (mV/dec)	Ref.	
MoS_2^{SO3H} (**4**)	H_2SO_4 0.1 M	302	68	This work	**Covalent**
	KOH 0.1 M	346	72		
Et_2NPh-MoS_2	H_2SO_4 0.5 M	348	75	1	
MoS_2-PhCN	H_2SO_4 0.5 M	251	120	2	
nBu-MoS_2	H_2SO_4 0.5 M	169	62	3	
APTES-MoS_2	H_2SO_4 0.5 M	382	110	4	**Not-covalent**
Im-MoS_2	H_2SO_4 0.5 M	283	102	5	
EDA-MoS_2	H_2SO_4 0.5 M	354	73	6	
BMI-MoS_2	H_2SO_4 0.5 M	362	62		

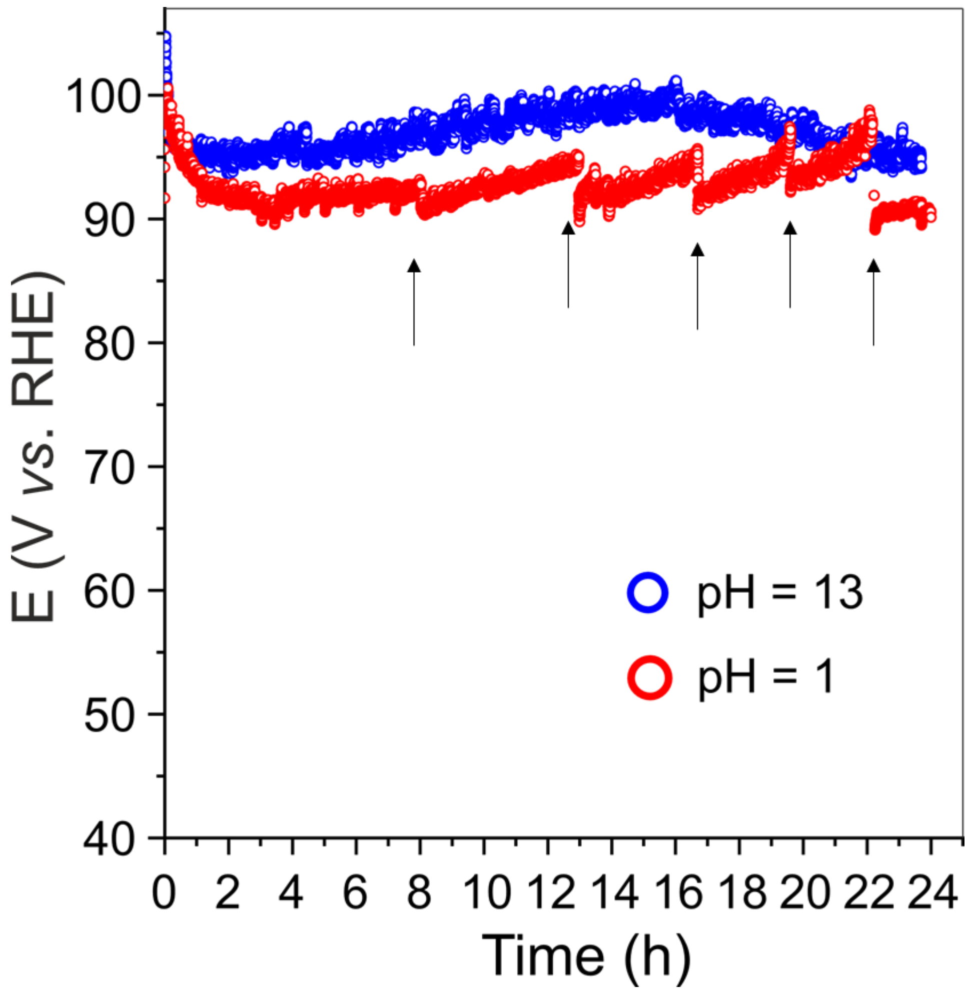

Figure S7. Durability tests for MoS_2^{SO3H} (**4**) conducted for 24 h in acidic and alkaline conditions. Due to the small volume of the cell (50 mL), during the 24 hours chronopotentiometry a refill of 10 mL of pre-degassed electrolyte was operated periodically (see black arrows) and till the end of the experiment. The unstable current is due to periodic accumulation and release of bubbles from the electrode surface.

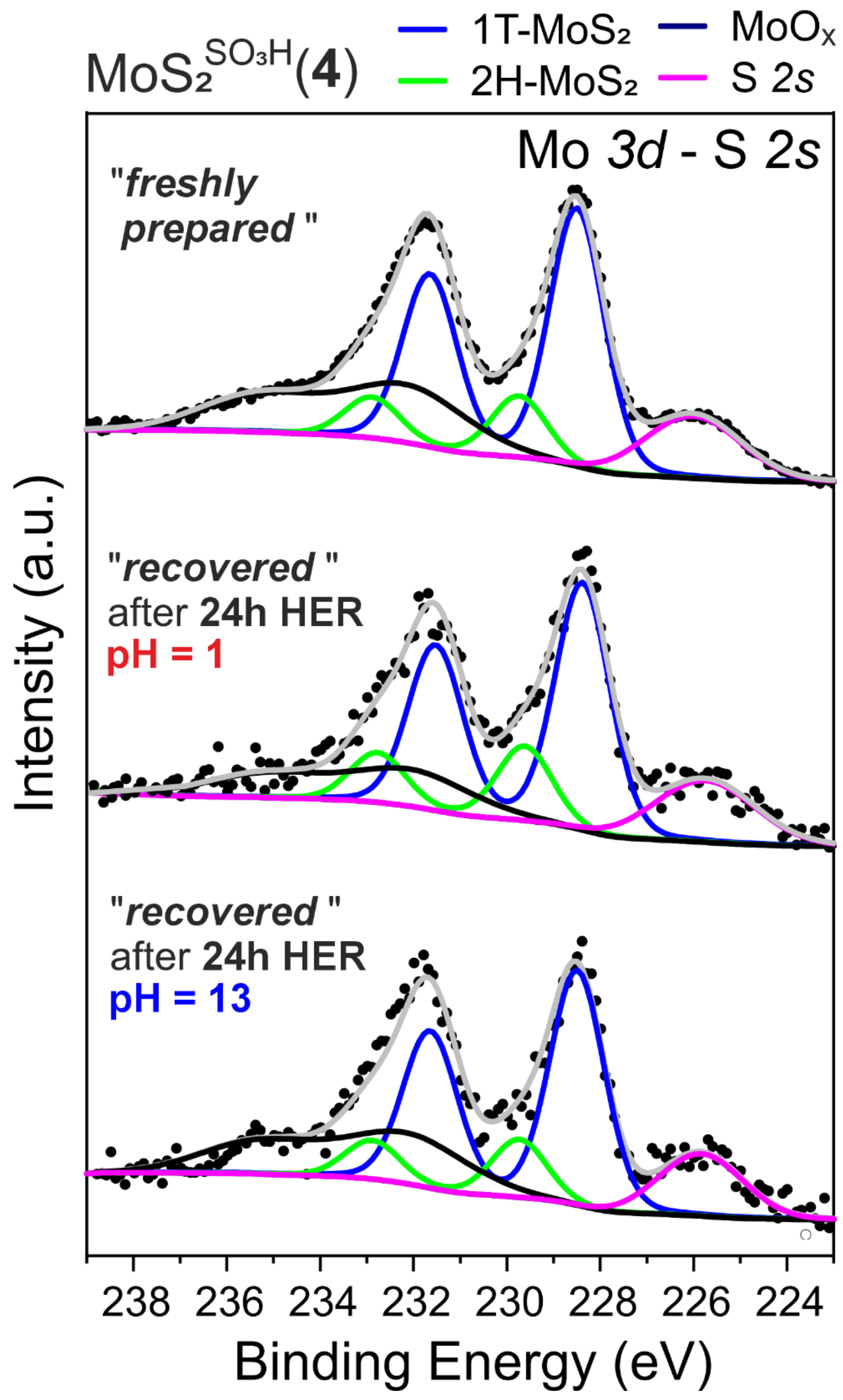

Figure S8. High resolution Mo *3d* XPS spectra of MoS_2^{SO3H} (**4**) recovered after HER electrocatalytic tests.

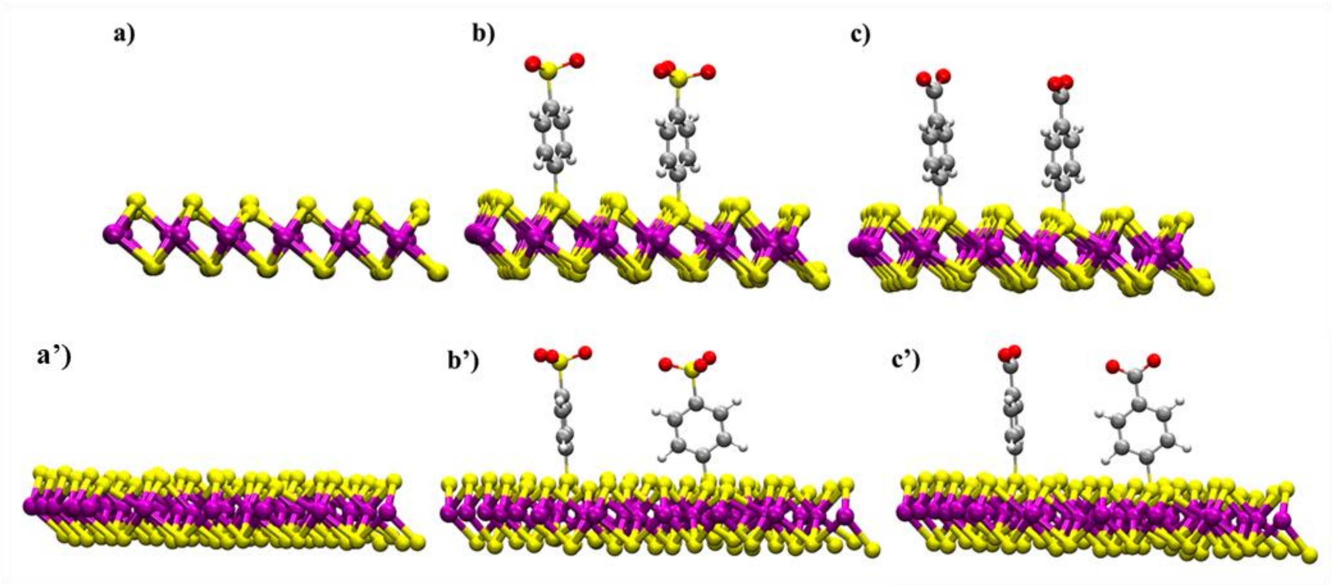

Figure S9. Optimized structure of the stochiometric a) 1T-MoS_2, b) 1T-MoS_2@2$PhSO_3^-$ and c) 1T-MoS_2@2$PhCOO^-$ and their S-defective counterparts a') 1T-$MoS_{2,1Vs}$, b') 1T-$MoS_{2,1Vs}$@2$PhSO_3^-$ and c') 1T-$MoS_{2,1Vs}$@2$PhCOO^-$ catalysts. Mo, S, C, H and O atoms are represented in balls & sticks and depicted in purple, yellow, black, white, and red, respectively.

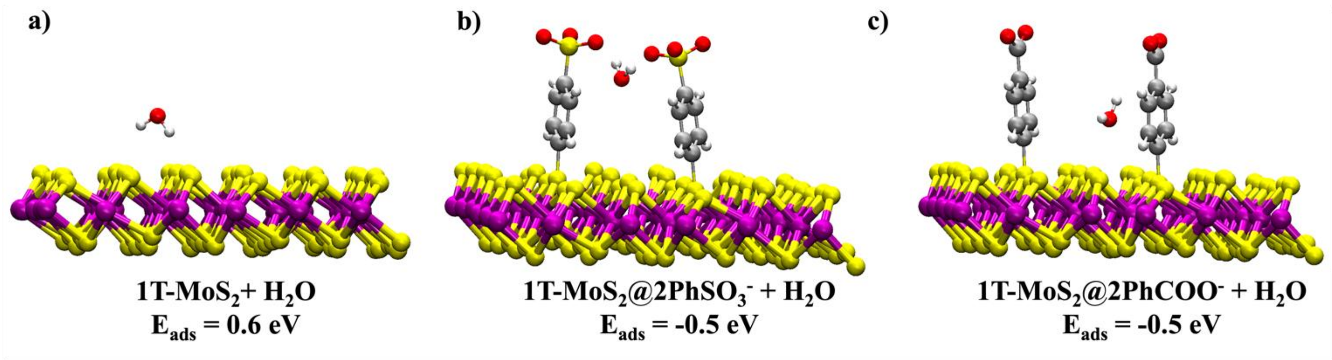

Figure S10. Optimized geometries of the H_2O molecule adsorption on the stochiometric surfaces with the corresponding adsorption energy values (E_{ads}) in eV: a) 1T-MoS_2 + H_2O, b) 1T-MoS_2@2$PhSO_3^-$ + H_2O and c) 1T-MoS_2@2$PhCOO^-$ + H_2O. Mo, S, C, H and O atoms are represented in balls & sticks and depicted in purple, yellow, silver, white and red, respectively.

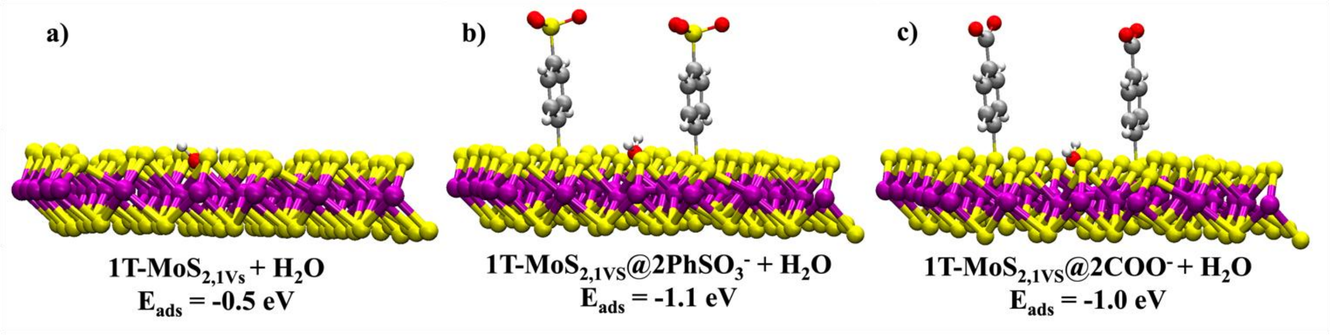

Figure S11. Optimized geometries of the H_2O molecule adsorption on the S-defective surfaces with the corresponding adsorption energy values (E_{ads}) in eV: a) $1T\text{-}MoS_{2,1Vs} + H_2O$, b) $1T\text{-}MoS_{2,1Vs}@2PhSO_3^- + H_2O$ and c) $1T\text{-}MoS_{2,1Vs}@2PhCOO^- + H_2O$. Mo, S, C, H and O atoms are represented in balls & sticks and depicted in purple, yellow, silver, white and red, respectively.

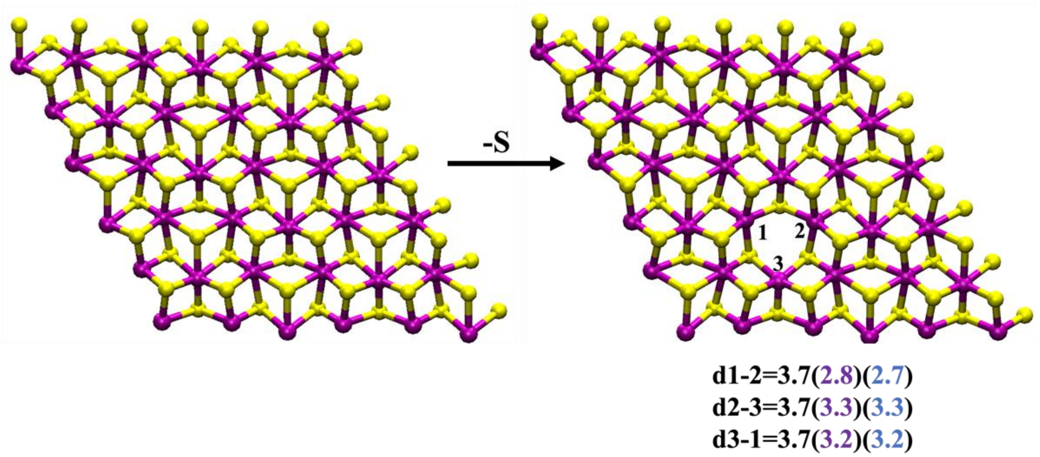

Figure S12. Top view of the optimized structure of the S-defective $1T\text{-}MoS_{2,1Vs}$ catalyst in which the Mo atoms (in purple) are exposed. The same Vs position is considered in $1T\text{-}MoS_{2,1Vs}@2PhSO_3^-$ and $1T\text{-}MoS_{2,1Vs}@2COO^-$. The exposed Mo atoms, deriving from Vs, are indicated with 1, 2 and 3. The distance between Mo1, Mo2 and Mo3 are in angstrom (Å) and reported in black for $1T\text{-}MoS_{2,1Vs}$, purple for $1T\text{-}MoS_{2,1Vs}@2PhSO_3^-$ and blue for $1T\text{-}MoS_{2,1Vs}@2PhCOO^-$. Mo and S atoms are represented in balls & sticks and depicted in purple and yellow, respectively.

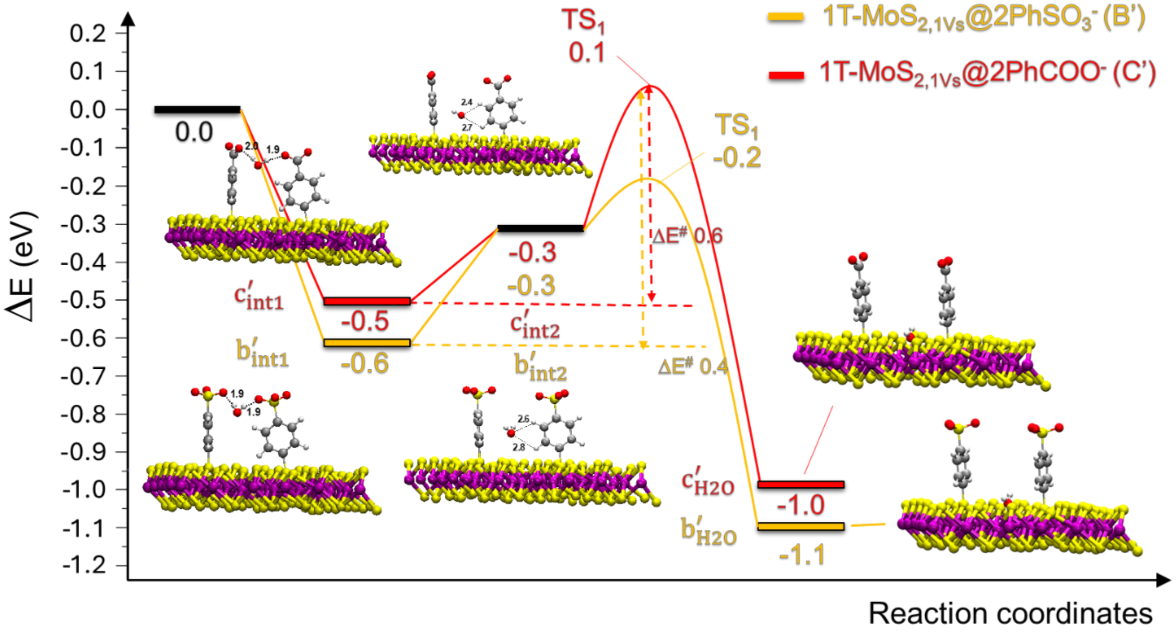

Scheme S1. Reaction pathways and activation energies for the H_2O tunnelling and adsorption at the Volmer step in alkaline environment on the catalysts 1T-$MoS_{2,1Vs}$@2$PhSO_3^-$ (orange line) and 1T-$MoS_{2,1Vs}$@2$PhCOO^-$ (red line) from climbing image nudged elastic band (CI-NEB) simulations along with their optimized structures. S, C, H and O atoms are represented in balls & sticks and depicted in purple, yellow, silver, white and red, respectively.

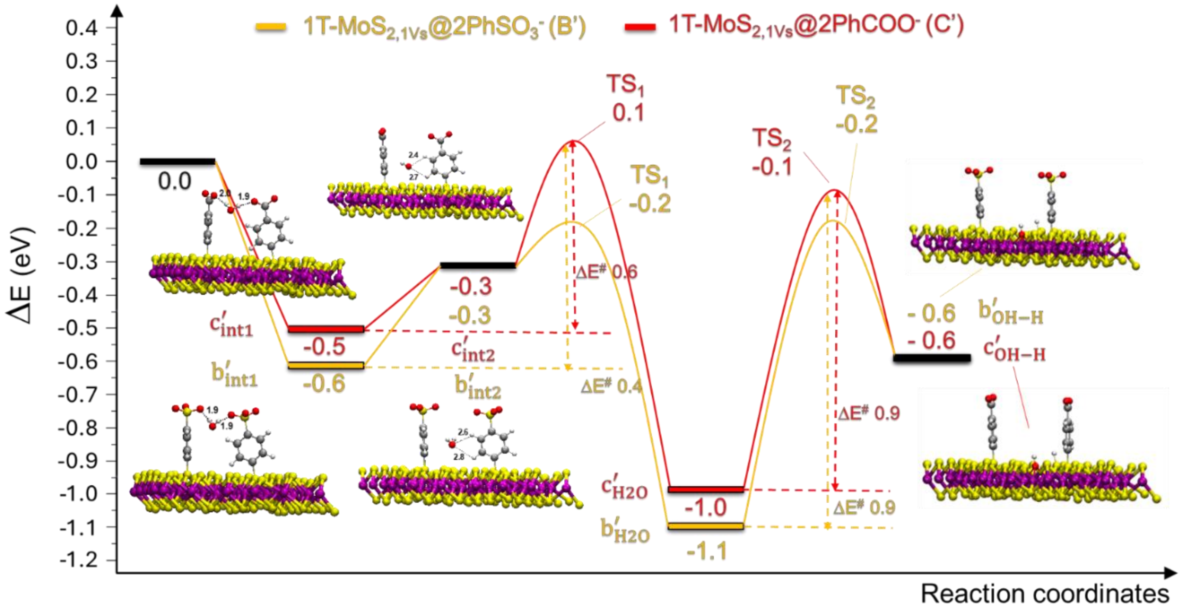

Scheme S2. Reaction pathways and activation energies for the H_2O tunnelling and dissociation at the Volmer step in alkaline environment on the catalysts 1T-$MoS_{2,1Vs}$@2$PhSO_3^-$ (orange line) and 1T-$MoS_{2,1Vs}$@2$PhCOO^-$ (red line) from climbing image nudged elastic band (CI-NEB) simulations along with their optimized structures. S, C, H and O atoms are represented in balls & sticks and depicted in purple, yellow, silver, white and red, respectively.

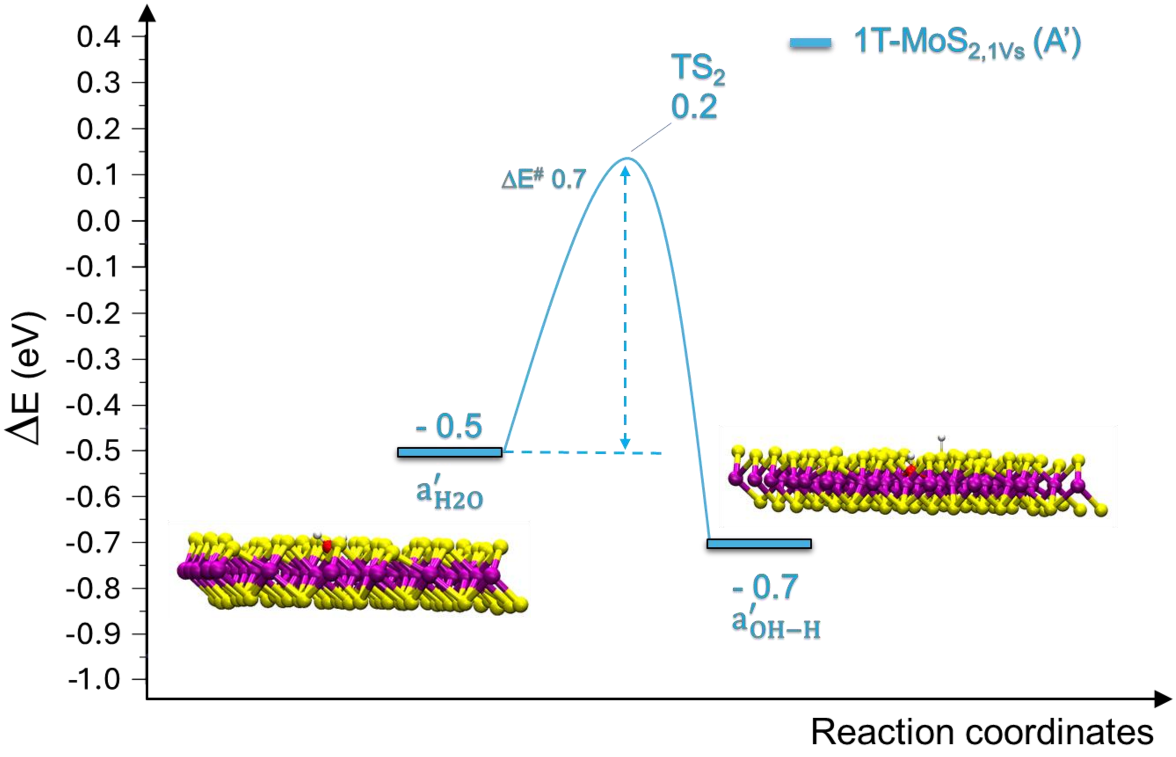

Scheme S3. Reaction pathway and activation energies for the H_2O tunnelling and dissociation at the Volmer step in alkaline environment on the catalyst 1T-$MoS_{2,1Vs}$ (A') from climbing image nudged elastic band (CI-NEB) simulations along with their optimized structures. The zero energy is free water and the catalyst 1T-$MoS_{2,1Vs}$ at infinite distance. Mo, S, H and O atoms are represented in balls & sticks and depicted in purple, yellow, white and red, respectively.

Scheme S1-3 details. For 1T-$MoS_{2,1Vs}$ (blue line), the first intermediate (a'_{H2O}), corresponding to the H_2O adsorption, lies at -0.5 eV with respect to the zero energy, where free water and the catalyst 1T-$MoS_{2,1Vs}$ are set at infinite distance. The water dissociation product (a'_{OH-H}), in which the adsorbed species H* and *OH are generated, is at -0.7 eV from the reactants and it is formed via the transition state TS_2 at 0.2 eV, overcoming an energy barrier of 0.7 eV.

Moving to the two functionalized sulfur-defective systems, the most stable geometries show the aromatic ring of the organic ligands oriented towards the sulfur vacancy (Vs), forming a stable interaction between

the exposed molybdenum (Mo) and a hydrogen atom of the aryl moiety of the ligand in the meta position relative to the $-SO_3^-$ and $-COO^-$ groups (see Figure S14). For the interaction with water to occur, the H-Mo interaction must be displaced by the incoming water molecule, which adsorbs onto the exposed metal site through its oxygen atom. Therefore, we started from 1T-$MoS_{2,1Vs}$@2PhSO_3^- and 1T-$MoS_{2,1Vs}$@2PhCOO$^-$ oriented towards Vs + H_2O at infinite distance (zero energy reference) (orange and red lines in Scheme S1 for 1T-$MoS_{2,1Vs}$@2PhSO_3^- and 1T-$MoS_{2,1Vs}$@2PhCOO$^-$, respectively). We found an initial reaction intermediate at -0.5 eV and -0.6 eV for 1T-$MoS_{2,1Vs}$@2PhCOO$^-$ and 1T-$MoS_{2,1Vs}$@2PhSO_3^-, respectively, stabilized by interactions between the hydrogen atoms of the water molecule and the oxygen atoms of the aromatic portion of the ligands (see intermediates c'_{int1} and b'_{int1} in Scheme S1). Then, the water molecule moves toward the surface, forming a less stable intermediate at -0.3 eV, characterized by interactions between the hydrogen atoms of the aromatic ring and the oxygen atom of the H_2O molecule (see intermediates c'_{int2} and b'_{int2} in Scheme S1). From these intermediates, the rotation of the ligands and the concerted interaction of H_2O with the sulfur vacancy (Vs) via TS_1 leads to water adsorption on the surface, with Eads of approximately -1.0 eV for both Brønsted-acids (see b'_{H2O} and c'_{H2O} in Scheme S1). The activation energy associated with ligand rotation is 0.4 eV for 1T-$MoS_{2,1Vs}$@2PhSO_3^- and 0.6 eV for 1T-$MoS_{2,1Vs}$@2PhCOO$^-$, suggesting a more difficult water adsorption on 1T-$MoS_{2,1Vs}$@2PhCOO$^-$ due to a higher energy barrier (of 0.2 eV) compared to 1T-$MoS_{2,1Vs}$@2PhSO_3^- . Finally, in both cases, the water dissociation products (b'_{OH-H} and c'_{OH-H}) are located 0.6 eV below the reactants. These are formed via TS_2 at -0.1 eV and -0.2 eV for 1T-$MoS_{2,1Vs}$@2PhCOO$^-$ and 1T-$MoS_{2,1Vs}$@2PhSO_3^-, respectively. Thus, for both ligands, the activation energy for H_2O dissociation is 0.9 eV (Scheme S2).

Although the energy trends related to the water dissociation at the Volmer step, as reported in Scheme S2, suggest that for the sulfur-defective 1T-MoS_2-based catalysts the rate-determining step is the dissociation of water, our calculations clearly indicate that the presence and nature of the organic ligands

make a significant contribution to the overall kinetics of the reaction: the presence of the aromatic pendants, interacting with the hydrogen atoms of H_2O, directs the water toward the channel formed by the two neighboring ligands, facilitating its approach to the surface (intermediates at -0.5 and 0.6 eV from the reactant at infinite distance). However, the vacancy interacts more readily with water only in the case of the Ar-SO_3^- pendant, as its weaker H-Mo interaction allows for a rapid rotation of the ring, making the metal site more available for stronger coordination of the water molecule onto the vacancy (0.5 eV more stable compared to the absence of the pendant). This enhanced coordination, in the case of 1T-$MoS_{2,1Vs}$@2PhSO_3^-, will facilitate the subsequent decisive dissociation step, in agreement with experimental data.

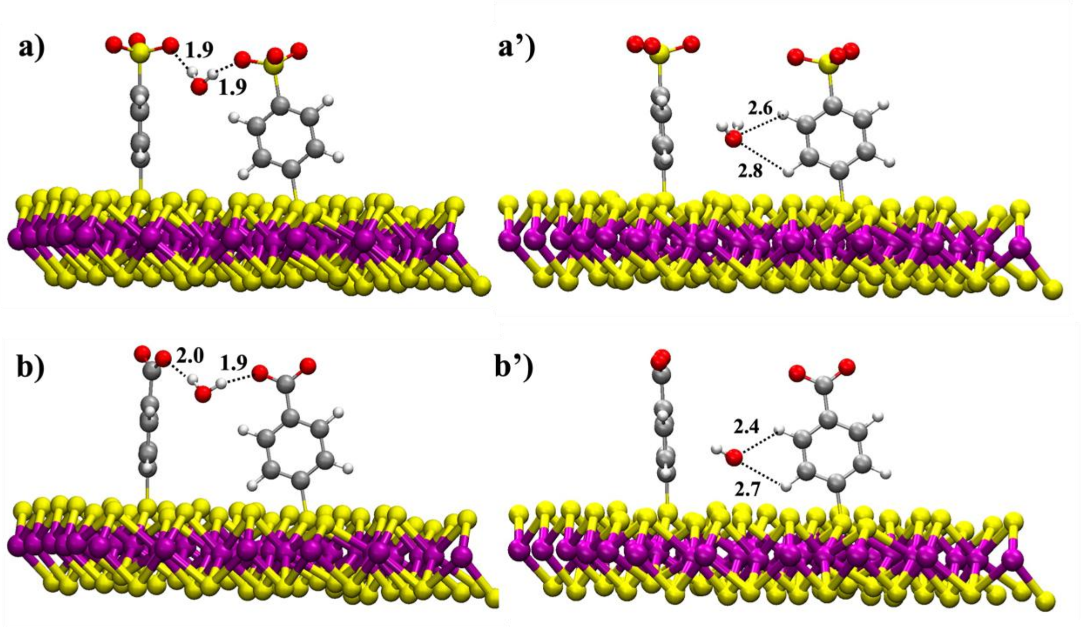

Figure S13. Intermediates b'$_{int1}$ and b'$_{int2}$ are shown in panels a) and a'), whereas intermediates c'$_{int1}$ and c'$_{int2}$ in panels b) and b'), respectively. The atomic distances are reported in Angstrom (Å). S, C, H and O atoms are represented in balls & sticks and depicted in purple, yellow, silver, white and red, respectively.

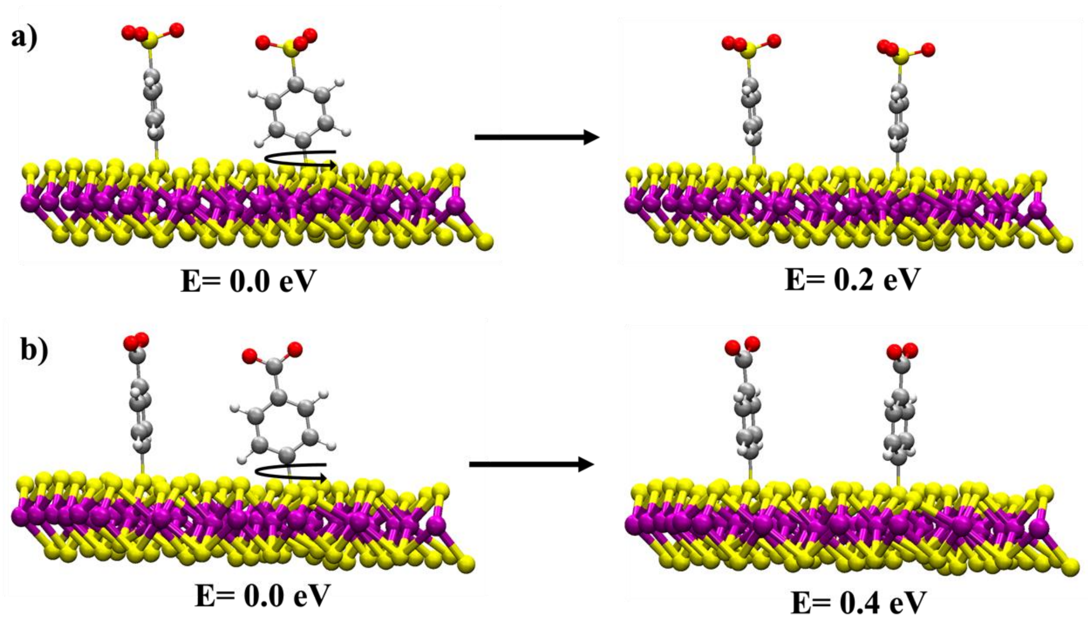

Figure S14. Energy differences in eV between the two possible geometries for a) 1T-$MoS_{2,1Vs}$@2$PhSO_3^-$ and b) 1T-$MoS_{2,1Vs}$@2$PhCOO^-$. S, C, H and O atoms are represented in balls & sticks and depicted in purple, yellow, silver, white and red, respectively.

References

1. Benson, E. E.; Zhang, H.; Schuman, S. A.; Nanayakkara, S. U.; Bronstein, N. D.; Ferrere, S.; Blackburn, J. L.; Miller, E. M., Balancing the Hydrogen Evolution Reaction, Surface Energetics, and Stability of Metallic MoS_2 Nanosheets via Covalent Functionalization. *J. Am. Chem. Soc.* **2018,** *140*, 441-450.

2. Cai, M.; Zhang, F.; Zhang, C.; Lu, C.; He, Y.; Qu, Y.; Tian, H.; Feng, X.; Zhuang, X., Cobaloxime anchored MoS_2 nanosheets as electrocatalysts for the hydrogen evolution reaction. *J. Mater. Chem. A* **2018,** *6*, 138-144.

3. Vedhanarayanan, B.; Shi, J.; Lin, J.-Y.; Yun, S.; Lin, T.-W., Enhanced activity and stability of MoS_2 through enriching 1T-phase by covalent functionalization for energy conversion applications. *Chem. Eng. J.* **2021,** *403*, 126318.

4. Pak, S.; Lim, J.; Hong, J.; Cha, S., Enhanced Hydrogen Evolution Reaction in Surface Functionalized MoS_2 Monolayers. *Catalysts* **2021,** *11*, 70.
5. Huang, W.-M.; Liao, W.-S.; Lai, Y.-M.; Chen, I.-W. P., Tuning the surface charge density of exfoliated thin molybdenum disulfide sheets via noncovalent functionalization for promoting hydrogen evolution reaction. *J. Mater. Chem. C* **2020,** *8*, 510-517.
6. Goloveshkin, A. S.; Lenenko, N. D.; Buzin, M. I.; Zaikovskii, V. I.; Naumkin, A. V.; Golub, A. S., Organic interlayers boost the activity of MoS_2 toward hydrogen evolution by maintaining high 1T/2H phase ratio. *Int. J. Hydrog. Energy* **2023,** *48*, 10555-10565.
